# Synergistic Effects of Toll-Like Receptor 1/2 and Toll-Like Receptor 3 Signaling Triggering Interleukin 27 Gene Expression in Chikungunya Virus-Infected Macrophages

**DOI:** 10.3389/fcell.2022.812110

**Published:** 2022-02-09

**Authors:** Juan Felipe Valdés-López, Geysson J. Fernandez, Silvio Urcuqui-Inchima

**Affiliations:** Grupo Inmunovirología, Facultad de Medicina, Universidad de Antioquia UdeA, Medellín, Colombia

**Keywords:** Interleukin 27, Chikungunya virus, toll-like receptors, antiviral response, NF-κB, pro-inflammatory response, innate immunity, transcriptomic

## Abstract

Chikungunya virus (CHIKV) is the etiological agent of chikungunya fever (CHIKF), a self-limiting disease characterized by myalgia and severe acute or chronic arthralgia. CHIKF is associated with immunopathology and high levels of pro-inflammatory factors. CHIKV is known to have a wide range of tropism in human cell types, including keratinocytes, fibroblasts, endothelial cells, monocytes, and macrophages. Previously, we reported that CHIKV-infected monocytes-derived macrophages (MDMs) express high levels of interleukin 27 (IL27), a heterodimeric cytokine consisting of IL27p28 and EBI3 subunits, that triggers JAK-STAT signaling and promotes pro-inflammatory and antiviral response, in interferon (IFN)-independent manner. Based on the transcriptomic analysis, we now report that induction of IL27-dependent pro-inflammatory and antiviral response in CHIKV-infected MDMs relies on two signaling pathways: an early signal dependent on recognition of CHIKV-PAMPs by TLR1/2-MyD88 to activate NF-κB-complex that induces the expression of EBI3 mRNA; and second signaling dependent on the recognition of intermediates of CHIKV replication (such as dsRNA) by TLR3-TRIF, to activate IRF1 and the induction of IL27p28 mRNA expression. Both signaling pathways were required to produce a functional IL27 protein involved in the induction of ISGs, including antiviral proteins, cytokines, CC- and CXC- chemokines in an IFN-independent manner in MDMs. Furthermore, we reported that activation of TLR4 by LPS, both in human MDMs and murine BMDM, results in the induction of both subunits of IL27 that trigger strong IL27-dependent pro-inflammatory and antiviral response independent of IFNs signaling. Our findings are a significant contribution to the understanding of molecular and cellular mechanisms of CHIKV infection.

## 1 Introduction

### 1.1 Chikungunya Virus

Chikungunya virus (CHIKV) is a zoonotic arthropod-borne pathogen, a member of *Alphavirus* genus, Togaviridae family, that can cause significant public health threats with significant social and economic impact (Silva and Dermody 2017). CHIKV is the etiological agent of chikungunya fever (CHIKF), a self-limiting disease characterized by the injury and inflammation of musculoskeletal and joint tissues. In humans, the acute phase of CHIKF lasts for 1–2 weeks. However, some disease symptoms, such as joint swelling, joint stiffness, arthralgia, arthritis, and tendonitis, can last for months to years in 10–50% of patients (Borgherini et al., 2008; Sissoko et al., 2009; Schilte et al., 2013).

CHIKF is associated with the development of immunopathology linked to high levels of pro-inflammatory cytokines, including tumor necrosis factor-alpha (TNFα), Interleukin (IL) 1β (IL1β), IL6, IL12p70, and IL15, both CC- and CXC-chemokines (CCL2, CCL3, CCL5, CCL8, CXCL9, CXCL10, and CXCL11), both in CHIKV-infected patients and *in vitro* culture (Wauquier et al., 2011; Dupuis-Maguiraga et al., 2012; Gasque et al., 2015; Valdés-López et al., 2019). Among the cytokines/chemokines, IL1β, IL6, CCL5, and CCL8 are correlated with the severity of CHIKF, while others, including IL1Ra, IL12p70, IL16, IL17, IL18, CCL2, and CXCL10, are correlated with high CHIKV loads (Chow et al., 2011; Dupuis-Maguiraga et al., 2012); Cavalcanti et al. (2019) reported that serum levels of IL27 were higher in patients with chronic CHIKF than in the ones with acute or subacute disease. Importantly, high levels of IL27 correlate with the persistence of CHIKV-dependent arthralgia.

CHIKV is primarily transmitted by bites of infected female mosquitoes of the *Aedes* genus. Then, CHIKV replicates in human epithelial cells, endothelial cells, fibroblasts, monocytes, and macrophages; subsequently, the virus enters lymph nodes and finally disseminates to other tissues *via* blood circulation (Sourisseau et al., 2007; Kam et al., 2009; Valdés-López et al., 2020).

### 1.2 Toll-Like Receptors

The first line of host defense following virus infection is the innate immune response through pattern recognition receptors (PRRs), as Toll-like receptors (TLRs) (Janeway and Medzhitov 2002). TLRs sense microbial components called pathogen-associated molecular patterns (PAMPs) to activate pro-inflammatory and antiviral responses (Aderem and Ulevitch 2000; Meylan et al., 2006; Lester and Li 2014). Both *in vivo* and *in vitro* studies suggest that innate immune response plays an important role in anti-viral defense by up-regulating the expression and activation of TLRs, which lead to the release of various inflammatory cytokines and immune modulators to promote virus clearance (Li et al., 2012; Priya et al., 2014; Valdés-López et al., 2020).

Among cell surface TLRs, TLR2 forms homodimer or heterodimer with TLR1 or TLR6 (Gay et al., 2014). These TLRs dimers play an essential role in sensing diverse microbial PAMPs, including lipoproteins, lipoteichoic acid (Gram-positive bacteria), and chitin (fungi) (Oliveira-nascimento et al., 2012). Several viruses have also been shown to interact with TLR2, including human immunodeficiency virus type 1 (HIV-1), Herpes simplex virus 1 (HSV1), Hepatitis C virus (HCV), Dengue virus (DENV), and severe acute respiratory syndrome coronavirus 2 (SARS-CoV-2) (Chang et al., 2007; Henrick et al., 2015; Brun et al., 2018; Aguilar-Briseño et al., 2020; Zheng et al., 2021). Upon recognition of PAMPs, TLR2 recruits adaptor proteins, myeloid differentiation primary response 88 (MyD88) and Interleukin 1 receptor-associated kinases (IRAK) to form the Myddosome, and initiate signal pathways that culminate in activation of nuclear factor-kB (NF-κB), and related transcription factors, including NF-κB1, NF-κB2, RELA, RELB, c-REL and IκBα (negative regulator) (Oeckinghaus and Ghosh 2009; Kawasaki and Kawai 2014). Activation of NF-κB complex results in the transcription of many NF-κB-target genes, including cytokines (TNFα, IL1β, IL6, and IL12p40) (Collart et al., 1990; Libermann and Baltimore 1990; Hiscott et al., 1993; Murphy et al., 1995), CC- chemokines (CCL17, CCL20, and CCL22) (Lee et al., 2008; Takegawa et al., 2008), CXC- chemokines (CXCL1 and CXCL8/IL8) (Kunsch and Rosen 1993; Ohtsuka et al., 1996), and enzymes such as cyclooxygenase 2 (COX2) (Collart et al., 1990), implicated in promoting pro-inflammatory response.

TLR3 on the other hand, recognizes intermediates of viral replication, such as double-strand RNA (dsRNA), and signals through adaptor protein TIR-domain-containing adapter-inducing interferon-β (TRIF), to activate signal pathways that culminate in the induction of NF-κB complex and interferon response factors (IRFs) (Okahira et al., 2005; Kawasaki and Kawai 2014). Both NF-κB and IRFs regulate the expression of pro-inflammatory mediators as well as interferons (IFNs) in many cell types (Okahira et al., 2005; Kawasaki and Kawai 2014). Susceptibility to CHIKV infection is markedly enhanced in human and mouse fibroblasts with defective TLR3 signaling or loss of TLR3, which aggravates CHIKV infection-related pathology (Her et al., 2015).

TLR4 is the only one among 10 TLRs described in humans that signals through both MyD88 and TRIF and acts as a heterodimer with CD14 or Lymphocyte antigen 96 (LY96) (Shen et al., 2008; Gay et al., 2014; Rajaiah et al., 2015). TLR4 plays an essential role in detecting lipopolysaccharide (LPS) from Gram-negative bacteria and induces robust pro-inflammatory response dependent on MyD88 and the activation of NF-κB (Kawasaki and Kawai 2014; Rajaiah et al., 2015).

### 1.3 Interferons

IFNs are a group of cytokines that regulate the immune system and induce an antiviral state in infected cells (Stetson and Medzhitov 2006). Type I IFN [IFN-I (IFNα, *β*, *ε*, *κ*, *ω*)] bind to IFN alpha/beta receptor complex [IFNAR (IFNAR1/IFNAR2)] and activate Janus kinase (JAK) signaling which phosphorylates and activates signal transducer and activator of the transcription 1 (STAT1) and STAT2 that, together with IRF9, are translocated into the nucleus and induce the expression of many IFN-stimulated genes (ISGs) (Taniguchi and Takaoka 2002; Piehler et al., 2012), that encode antiviral proteins (AVPs), cytokines, CC and CXC chemokines (Stetson and Medzhitov 2006; Valdés-López et al., 2021). In addition to IFNs, other cytokines could mediate activation of different components of JAK-STAT signaling, including members of IL2, IL6, IL10, and IL12 families (Ye et al., 2020; Salas et al., 2020).

### 1.4 Interleukin 27

IL27 is a heterodimeric cytokine, a member of the IL12 family of cytokines, composed of IL27p28 and Epstein-Barr virus-induced 3 (EBI3) subunits (Pflanz et al., 2002; Rousseau et al., 2010). IL27 signal through their interaction with IL27 receptor [IL27R (IL27Rα/Gp130)] on the cell surface, triggering the activation of JAK1/JAK2, which phosphorylates and activates STAT1/STAT3 transcription factors (Pflanz et al., 2004; Huber et al., 2007; Pradhan et al., 2007; Kwock et al., 2020). IL27 elicits both pro-and anti-inflammatory responses, although the later activity seems to be the dominant (Yoshida and Hunter 2015). Moreover, like IFNs, IL27 induces a strong antiviral response against HIV-1, Hepatitis B virus (HBV), HCV, Influenza A virus (IAV), and Zika virus (ZIKV) (Fakruddin et al., 2007; Frank et al., 2010; Liu et al., 2012; Cao et al., 2014; Kwock et al., 2020). These results are in agreement with our previous observation showing that IL27 signaling is activated in CHIKV-infected monocytes-derived macrophages (MDMs) and that the kinetics of IL27p28/EBI3 mRNA expression and IL27 protein production correlates with the expression of AVPs in CHIKV-infected MDMs, in an IFN-independent manner (Valdés-López et al., 2021). Furthermore, we showed that stimulation of THP-1-derived macrophages with recombinant-human IL27, activated JAK-STAT signaling and induced robust pro-inflammatory and antiviral response, comparable to CHIKV-infected MDMs. Taken together, our previous results show that CHIKV-infected MDMs expressed high levels of IL27 that activates JAK-STAT signaling and promotes pro-inflammatory and antiviral response to control CHIKV replication in an IFN-independent manner. However, a precise mechanism underlying IL27 expression in CHIKV-infected MDMs is unknown. Our objective here is to explore the molecular mechanisms that trigger IL27 gene expression in CHIKV-infected MDMs.

## 2 Material and Methods

### 2.1 Chikungunya Virus Stocks, Titration, and Ultraviolet Light Inactivation of Chikungunya Virus

A clinical isolate of CHIKV was obtained following the protocol described in (Pastorino et al., 2005) from a CHIKF patient (kindly gifted by Professor Francisco Javier Díaz, University of Antioquia). CHIKV was amplified from a Colombian patient’s serum and propagated in Vero cells (ATTC CCL-81), as we previously reported (Valdés-López et al., 2020) (Valdés-López et al., 2021). Briefly, cells were grown in Dulbecco’s Modified Eagle Medium (DMEM; Sigma-Aldrich, St. Louis, United States) supplemented with 5% heat-inactivated fetal bovine serum (FBS; Gibco, Thermo Fisher Scientific, Massachusetts, United States), 4 mM L-glutamine (Sigma-Aldrich), 0.3% (v/v) sodium carbonate (NaCO3; Sigma-Aldrich) and 1% (v/v) antibiotic-antimycotic solution (Corning-Cellgro, New York, United States), and incubated at 37°C and 5% CO_2_ in cell culture flasks, to a cell density of 1 × 10^5^-1 × 10^6^ cells/ml. Vero cells were inoculated with CHIKV at 0.1 multiplicity of infection (MOI), incubated at 37°C and 5% CO_2_ for 2 days, or until an advanced cytopathic effect was observed. Next, supernatants were collected, precleared by centrifugation (1,650 × g for 10 min), and stored at −80°C. CHIKV stocks were titrated by plaque assay on Vero cells, as previously described (Valdés-López et al., 2020). The virus titer was determined to be 2.1 × 10^8^ PFU/ml. CHIKV inactivation using ultraviolet (UV) radiation was performed by exposing 200 µL volumes of virus preparation at 10 cm of UV light (∼365 nm, 60 min, ∼145 mW/cm^2^) as was described in (Valdés-López et al., 2020). Inactivated CHIKV was stored to −80°C. The inactivation efficacy was confirmed by plaque assay in Vero cells and the plaques were counted and expressed as plaque-forming units per mL (PFU/ml).

### 2.2 Culture of Human Monocytes and Differentiation Into Monocytes-Derived Macrophages

The study was approved by the Ethics Committee of “Sede de Investigación Universitaria-Universidad de Antioquia”. Written informed consent was obtained from all individuals who voluntarily participated in this study, according to the principles expressed in the Declaration of Helsinki.

Human peripheral blood mononuclear cells (PBMCs) from blood samples of healthy donors mixed with 2% EDTA, were isolated through density gradient with Lymphoprep (STEMCELL Technologies Inc., Vancouver, Canada) by centrifugation (850 × g for 21 min) as previously described (Valdés-Lopéz et al., 2020). PBMCs from each healthy volunteer were prepared independently. Platelet depletion was performed by washing with PBS-1X (Sigma-Aldrich) three times at 250 × g for 10 min and the percentage of CD14 positive cells was determined by flow cytometry. To obtain monocytes, 24-well plastic plates were scratched with a 1,000 μL pipette tip and seeded with 5 × 10^5^ CD14 positive cells per well and allowed to adhere for 2 h in RPMI-1640 medium supplemented with 0.5 (v/v) autologous serum or plasma (to favor adherence of monocytes to the well), 4 mM L-glutamine and 0.3% (v/v) NaCO_3_, and cultured at 37°C and 5% CO_2_. Non-adherent cells were removed by washing twice with PBS-1X and monocytes were cultured in RPMI-1640 medium supplemented with 10% (v/v) FBS, 4 mM L-glutamine, 0.3% (v/v) NaCO3, and 1% (v/v) antibiotic-antimycotic solution 100X (complete medium), and incubated at 37°C and 5% CO_2_ for 6 days to obtain MDMs, as previously described (Valdés-Lopéz et al., 2020; Valdés-López et al., 2021).

### 2.3 *In vitro* Chikungunya Virus Infection of Monocytes-Derived Macrophages

Infection of human primary MDMs was performed with CHIKV or with UV-Inactivated CHIKV (UV-CHIKV) at MOI 5 in serum-free RPMI-1640 medium as previously reported (Valdés-López et al., 2020). Samples were incubated at 37°C for 1.5 h. An hour and a half after infection, the cells were washed with PBS-1X to remove the unbound virus and fresh complete medium was added and incubated at 37°C with 5% CO_2_. Culture supernatants and cell lysates were collected at 6, 24, and 48 hpi and stored at −80°C.

### 2.4 RNA Extraction and cDNA Libraries Synthesis

Total RNA from uninfected or CHIKV-infected MDMs was obtained using Direct-zol™ RNA Miniprep Plus (Zymo Research, Irvine, California) following the manufacturer’s protocol. RNA samples were treated with DNase I column (Zymo Research, Irvine, California) to remove contaminating genomic DNA. RNA was quantified by spectrophotometry (Thermo Scientific, Wilmington, DE, United States). cDNA libraries were constructed for each experimental group using the RevertAid Minus First Strand cDNA Synthesis Kit (Thermo Scientific), according to the manufacturer’s instructions. The samples were stored at −80°C. One μg of RNA was used for RNA-Seq, and 400 ng for cDNA synthesis.

### 2.5 RNA-Sequencing Data and Bioinformatics Analysis

RNA sequencing was carried out on Illumina NextSeq 550 platform. After sequencing, image data was transformed into raw reads and stored in FASTQ datasets for each sample, using FastQC (google/vFqiZ) as we described in (Fernandez et al., 2020). Clean reads were obtained by removing the low-quality adapter, poly-N containing, and shorter-than-70 bp reads and mapped to the human transcriptome (RefSeq, HG38) using TopHat software (version 1.3.2.). The HTseq software for Python was used to generate counts for each gene from the mapped sequences. Counts matrix was normalized to transcripts per million (TPM). Then, gene expression (mRNA) was normalized by calculating Reads per kilobase per million mapped reads (RPKM) [TPM/Gene size (Kbs)]. Differential expression of mRNAs in each experimental group was identified using the DEseq package (version 1.8.261) implemented in R software (version 3.6.3). To determine the differentially expressed genes (DEG), we used the edgeR package of R software where the false discovery rate (FDR) < 0.05 and the |Log2 Fold Change (FC) (CHIKV-infected MDMs/Uninfected MDMs) |> 0.6 (|log2FC|> 0.6), were used as the threshold to determine the statistically significant difference in gene expression. Gene Ontology (GO) was performed with the BiNGO Cytoscape plugin, using a hypergeometric test with a Benjamini and Hochberg False Discovery Rate correction to identify significant functions of the DEG. A *p* < 0.05 and a Fold-Enrichment >4.0 were used to identify enriched processes.

### 2.6 Transcription Factor Binding Motifs Analysis

HOMER (Hypergeometric Optimization of Motif EnRichment) (v4.11; http://homer.ucsd.edu/homer/motif/) (Heinz et al., 2010) was used to identify Transcription factor binding motifs (TFBM) overrepresented in the promoter of the human IL27p28 and EBI3 genes as was described before (Fernandez et al., 2020). Gene promoters were considered between nucleotides -600 and +50 relative to the Transcription Start Site (TSS). Significance was tested against CpG-content-matched promoters as background. Binding sites were considered significantly overrepresented when the Score> 3, and *p*-value <0.01.

### 2.7 Differential Expression Analysis of Previously Published Data

To confirm our results concerning the NF-κB-complex activation observed in CHIKV-infected MDMs, we reanalyzed the publicly available RNA-seq GSE84188 (GEO) (Olejnik et al., 2017) and the microarray GSE89962 (GEO) (Lin et al., 2017). The first RNA-seq dataset was performed from human MDMs stimulated with 100 ng/ml of LPS, an agonist for TLR4, and a recognized inductor of NF-κB-complex activation via MyD88 (Küper et al., 2012; Sakai et al., 2017), for 6, 24 or 48 h (Olejnik et al., 2017). To define the top DEG, we selected genes with an FDR< 0.05 and |Log2 Fold Change (LPS-stimulated MDMs/unstimulated MDMs) |> 0.6. Differential expression of mRNAs was reanalyzed as described in the RNA-seq data and bioinformatics analysis section.

The microarray dataset (GSE89962) was obtained from Bone marrow-derived macrophages (BMDM) of C57BL/6 mice stimulated with 10 ng/ml Pam3CSK4 and/or 10 μg/ml Poly (I:C), or 10 ng/ml LPS (TLR1/2, TLR3, and TLR4 agonists, respectively), for 8 h (Lin et al., 2017). Differential expression of mRNAs in each experimental group was identified using GEO2R. To define the top DEG, we selected genes with an FDR< 0.05 and |Log2 Fold Change (TLR-treated BMDM/unstimulated BMDM) |> 0.6.

### 2.8 Activation of Toll-Like Receptors in Human Monocytes-Derived Macrophages

Human MDMs were stimulated with 50 ng/ml Pam3CSK4 and/or 20 μg/ml Poly (I:C), or 50 ng/ml of ultrapure LPS, agonists for TLR1/2, TLR3, and TLR4, respectively, in fresh complete medium and incubated at 37°C and 5% CO_2_ for 8 h. All TLR agonists were obtained from InvivoGen (San Diego, CA, United States). Culture supernatants and cell lysates were collected and stored at −80°C.

### 2.9 Validation of mRNA-Seq Results by Real-Time Quantitative PCR

The RT-qPCR method was used to validate the expression of key genes obtained with the analysis of two RNA-Seq (CHIKV-infected MDMs and LPS-stimulated MDMs) and MicroArray performed in murine BDMD treated with TLR agonists. First-strand cDNA was generated using SuperScript III reverse transcriptase and specific primer pairs were used to quantify IL27p28, EBI3, IFNβ1, OAS1, PKR, TLR2, TLR3, and B-Actin as previously reported (Valdés-Lopéz et al., 2020; Valdés-López et al., 2021). Brief, PCR amplifications were carried out using the SYBR system (Invitrogen, Oregon, United States). The Bio-Rad CFX manager was used to obtain the cycle thresholds (Ct) that were determined for each sample using a regression fit in the linear phase of the PCR amplification curve. Relative expression of each target gene was normalized to the unstimulated control and housekeeping gene *β*-actin (ΔΔCt) and was reported as the Log2 Fold Change.

### 2.10 Cytokine Quantification

The ELISA MAX™ Deluxe Set for Human TNFα,IL6, MCP-1/CCL2 and CCL5 (RANTES) (BioLegend, San Diego, CA, United States) was used for detection of TNFα,IL6, MCP-1/CCL2 and CCL5 proteins in supernatants of MDMs cultures, following manufacturer´s instructions. The detection limit was 2–4 pg/ml. The LEGEND MAX™ Human IL27 ELISA Kit (BioLegend, San Diego, CA, United States) was used for the detection of mature IL27 protein in supernatants of MDMs cultures, following the manufacturer’s instructions. The detection limit was 11 pg/ml.

### 2.11 *In vitro* Antiviral Assay

Human MDMs were pre-treated for 8 h with 50 ng/ml Pam3CSK4 and/or 20 μg/ml poly (I:C), or 50 ng/ml of ultrapure LPS. Further, MDMs were treated with 25 ng/ml of recombinant-human IL27 (BioLegend) or 25 ng/ml of recombinant-human IFNβ1 (STEMCELL Technologies Inc.), which were used as the positive control. Next, MDMs were infected with CHIKV at MOI 5 in serum-free RPMI-1640 medium. Samples were incubated at 37°C for 1.5 h. Then, the cells were washed with PBS-1X to remove the unbound virus and a fresh complete medium was added and incubated at 37°C with 5% CO_2_. Culture supernatants were obtained at 24 hpi and stored at −80°C. CHIKV was titrated by plaque assay on Vero cells.

### 2.12 Statistical Analysis

Statistical analysis was performed using GraphPad Prism 5 (GraphPad Software Inc. San Diego, CA). Statistical tests are indicated in the figure legends. Data are represented as mean ± SEM, Log2 Fold Change (Log2 FC), or RPKM. Significant results were defined as *p* < 0.05 (*) (#), *p* < 0.01 (**) (##) and *p* < 0.002 (***) (###).

## 3 Results

### 3.1. IL27p28 and EBI3 mRNAs Have Different Expression Kinetics in Chikungunya Virus-Infected Monocytes-Derived Macrophages

**FIGURE 1:**
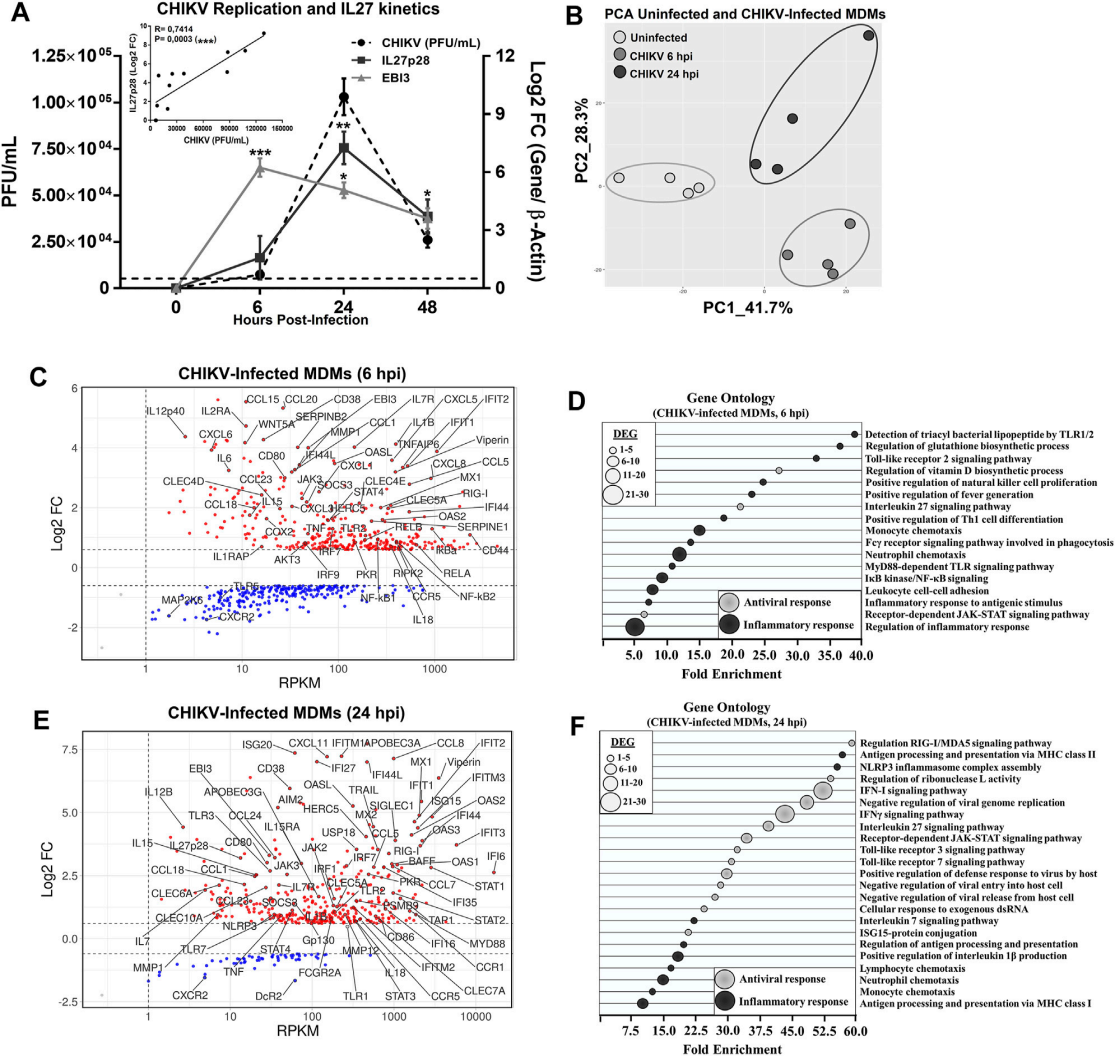
CHIKV infection induces the expression of both subunits of IL27 and activated a dynamic transcriptional profile in human MDMs. Human MDMs cultures were left uninfected or infected with CHIKV at MOI 5. Culture supernatants were obtained at 6, 24, and 48 hpi, and quantification of CHIKV PFU/mL was performed by plaque assay on Vero cells. MDMs lysates were obtained at 6, 24, and 48 hpi, and RT-qPCR was performed. CHIKV replication, IL27p28 and EBI3 mRNA expression kinetics in human MDMs **(A)**. Data are presented as the mean ± SEM. Mann-Whitney test was performed. Significant results are defined as *p* < 0.05 (*), *p* < 0.01 (**) and *p* < 0.002 (***). *n* = 4. Pearson’s correlation between CHIKV replication (PFU/ml) and IL27p28 mRNA expression (Log2 FC) in CHIKV-infected MDMs **(A)**. Significant results are defined as *p* < 0.05 (*), *p* < 0.01 (**) and *p* < 0.002 (***). Human MDMs cultures were left uninfected or infected with CHIKV at MOI 5. Cell lysates were obtained at 6 and 24 hpi and RNA-seq was performed. PCA plot **(B)** of differentially expressed genes (DEG) interrogated by RNA-Seq. MA plots **(C and E)** and GO enrichment analysis **(D and F)** from DEG regulated in CHIKV-infected MDMs at 6 and 24 hpi, respectively. DEG in CHIKV-infected MDMs was selected from genes with an FDR< 0.05 and |Log2 Fold Change (CHIKV-infected MDMs/Uninfected MDMs) |> 0.6. To quantify mRNAs abundance, we use the Reads per kilobase per million mapped reads (RPKM). *n* = 4.

We previously reported (Valdés-Lopéz et al., 2020; Valdés-López et al., 2021) that CHIKV replication in human MDMs is fast, with a peak of infectious viral particles at 24 hpi and a significant decline at 48 hpi. The focus of the present study is the expression of IL27 subunits in CHIKV infected human MDMs. As shown in Figure 1A, CHIKV infection of MDMs induced IL27p28 and EBI3 mRNA expression with differential kinetics. While IL27p28 reaches its peaked expression at 24 hpi, which correlated with the peak of CHIKV replication (*R* = 0.7414; *p* = 00,003), the peak of EBI3 mRNA expression was observed at 6 hpi (Figure 1A). The results suggest that the expression of IL27 subunits may be differentially regulated in CHIKV-infected MDMs.

### 3.2 Chikungunya Virus Infection Induces a Dynamic Transcription Pattern in Human Monocytes-Derived Macrophages

To explorer the transcriptional dynamics of the host, including expression of IL27 subunits and related genes in response to CHIKV infection in human MDMs, we performed RNA-seq analysis of uninfected and CHIKV-infected MDMs at 6 and 24 hpi. We analyzed sample variance in RNA-Seq dataset using principal component analysis (PCA) (Figure 1B). According to the PCA, the first principal component (PC1), which was able to discriminate uninfected MDMs from CHIKV-infected MDMs, showed high heterogeneity in mRNAs profiles of around 41.7% expression variance. PC2 revealed distinct expression profiles of uninfected MDMs from CHIKV-infected MDMs, representing 28.3% of total expression variance between samples due to the response of MDMs to viral infection. Further, our data showed that CHKV-infected MDMs have a different transcriptional profile at 6 and 24 hpi (Figures 1C,E, respectively). To define the top DEG, we selected genes with an FDR< 0.05 and |Log2 FC (CHIKV-infected MDMs/Uninfected MDMs) |> 0.6. Of the 60,592 genes interrogated (by RNA-Seq), 361 and 259 were respectively up or down-regulated at 6 hpi (in CHIKV-infected MDMs) (Figure 1C). Up-regulated genes include TLRs and components of TLR signaling pathway (TLR1, TLR2, and IRAK2), pro-inflammatory factors, such as TNFα, IL1β, IL6, IL12p40; both CC-and CXC-chemokines, including CCL3, CCL5, CCL15, CCL20, CXCL1, CXCL5, CXCL6, and CXCL8/IL8, and components of NF-κB-complex (NF-κB1, NF-κB2, RELB, and IκBα). The result suggests that at 6 hpi, in addition to inducing the expression of EBI3 (Figure 1C), CHIKV-infected MDMs trigger a robust pro-inflammatory response. Among the down-regulated genes (at 6 hpi) include TLR5, CXCR2, and MAP2K6 (Figure 1C). GO functional analysis of DEG was performed to gain an insight into the biological roles of the most significantly up-regulated genes in CHIKV-infected MDMs at 6 hpi (Figure 1D). DEGs were enriched for biological processes associated with the induction of a robust pro-inflammatory program, including detection of triacyl bacterial lipopeptide by TLR1/2, Toll-like receptor 2 signaling pathway, MyD88-dependent TLR signaling pathway, IκB kinase/NF-κB signaling, positive regulation of fever generation, monocyte and neutrophil chemotaxis, and others, suggesting that modulation of these genes may be essential for the induction of an early pro-inflammatory response in CHIKV-infected MDMs.

At 24 hpi, 367 and 61 genes were up or down-regulated, respectively, in CHIKV-infected MDMs (Figure 1E). The up-regulated genes include components of TLR signaling pathway (TLR3, TLR7, and MyD88), IL27 signaling pathway (Gp130, JAK2, STAT1, STAT3, and SOCS3), and ISGs mRNA encoding AVPs [Apolipoprotein B Editing Complex 3 (APOBEC3) family of proteins, Guanylate-binding proteins (GBP), interferon-inducible (IFI) family of proteins, interferon-induced transmembrane (IFITM) proteins, ISG15, ISG20, dynamin-like GTPase 1 (MX1), MX2, oligoadenylate synthase (OAS) family of proteins, double-stranded RNA-activated protein kinase R (PKR), Viperin, and others], associated with induction of antiviral state and control of CHIKV replication in MDMs [A complete analyses of antiviral response in CHIKV-infected MDMs at 24 hpi was presented in (Valdés-López et al., 2021)]. The down-regulated genes at 24 hpi include DcR2 and CXCR2 (Figure 1E). GO functional analysis of DEGs were performed to gain an insight into the biological roles of the most significantly up-regulated genes in CHIKV-infected MDMs at 24 hpi (Figure 1F). DEGs were enriched for biological processes associated with the induction of a robust antiviral program, including regulation of RIG-I/MDA5 signaling pathway, TLR3, and TLR7 signaling pathway, IFN-I and IFNγ signaling pathway (grouped as a high diversity of AVPs and inflammatory molecules), IL27 signaling pathway, receptor-dependent JAK-STAT signaling pathway, and others, suggesting that modulation of these genes may be essential for the control of CHIKV replication in MDMs.

### 3.3 Transcription of Genes Encoding IL27p28 and EBI3 Are Differentially Regulated

**FIGURE 2:**
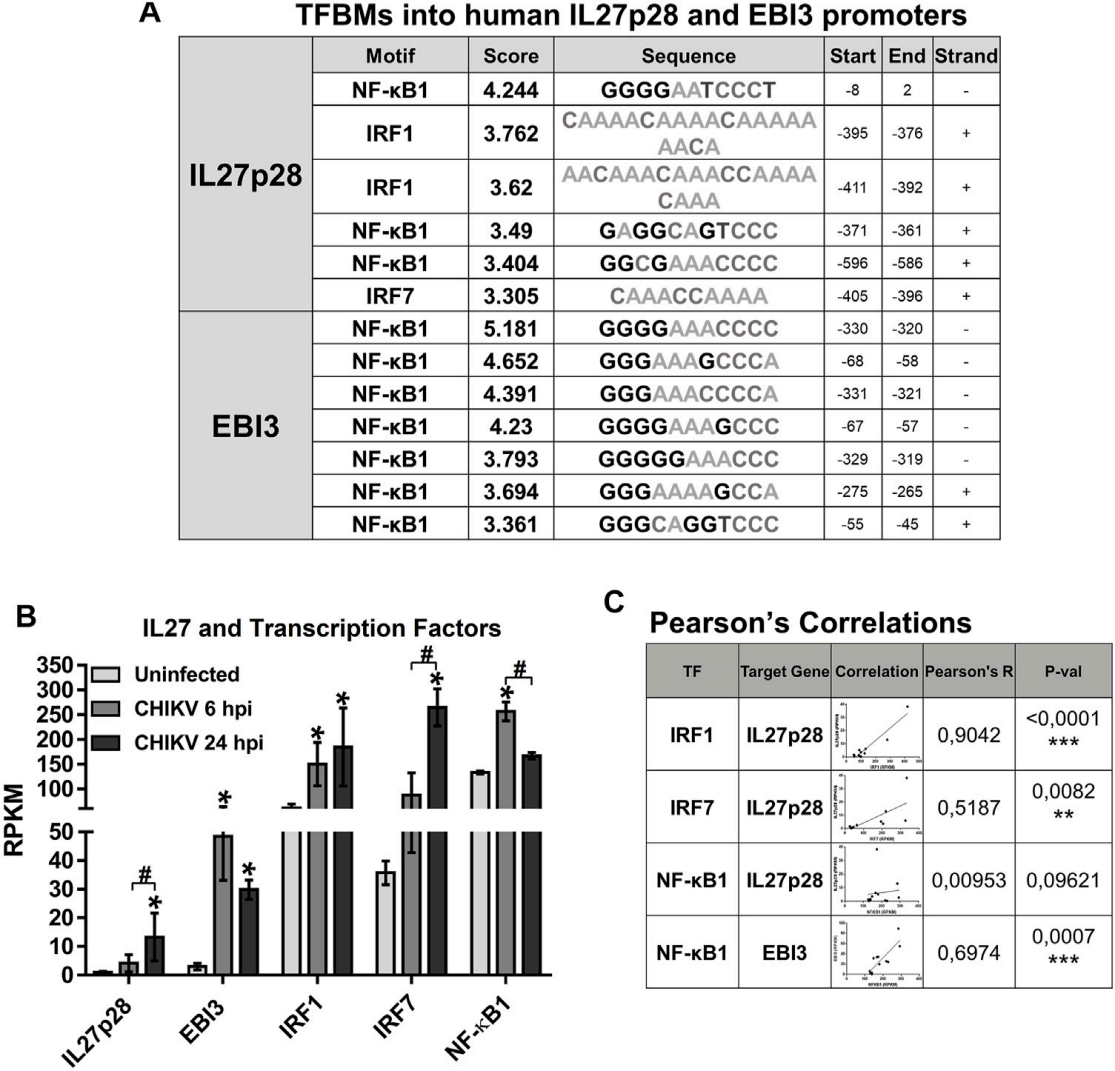
The human encoding genes for IL27p28 and EBI3 have differential transcriptional regulation. TFBM analysis was performed on promoters (−600 and +50 relative to TSS) of human IL27p28 and EBI3 genes using HOMER software. TFBM on the promoter of human IL27p28 and EBI3 genes **(A)**. Human MDMs cultures were left uninfected or infected with CHIKV at MOI 5. Cell lysates were obtained at 6 and 24 hpi and RNA-seq was performed. mRNAs abundance (RPKM) of IL27 subunits (IL27p28 and EBI3) and transcription factors (IRF1, IRF7 and NF-κB1) in uninfected and CHIKV-infected MDMs **(B)** Data are presented as the mean ± SEM. Kruskal–Wallis test with Dunn’s post-test was performed. Significant results between uninfected and CHIKV-infected MDMs are defined as *p* < 0.05 (*), *p* < 0.01 (**) and *p* < 0.002 (***). *n* = 4. Pearson’s correlation between transcription factors (IRF1, IRF7 and NF-κB1) and IL27 subunits (IL27p28 and EBI3) mRNAs abundance (RPKM) in uninfected and CHIKV-infected MDMs **(C)**. Significant results are defined as *p* < 0.05 (*), *p* < 0.01 (**) and *p* < 0.002 (***).

To understand the regulatory mechanisms of IL27p28 and EBI3 transcription in CHIKV-infected MDMs, we searched for transcription factor binding motifs (TFBM) present in the promoter regions of human IL27p28 and EBI3 genes (Figure 2A). We found that IL27p28 promoter sequence includes motifs for IRF1, IRF7, and NF-κB1 binding, while EBI3 promoter possessed only NF-κB1 binding sites, suggesting that transcriptional regulation of the two IL27 subunits is different. All those transcription factors were also expressed in MDMs infected with CHIKV at 6 and 24 hpi (Figure 2B). Furthermore, we observed that IL27p28 mRNA expression had significant positive correlation with IRF1 mRNA (*R* = 0.904, *p* < 0.001), and a moderate but significant positive correlation with IRF7 mRNA (*R* = 0.5187, *p* = 00,082), with a high expression at 24 hpi (Figure 2B). Moreover, we noted a negligible correlation of IL27p28 mRNA with NF-κB1 mRNA (*R* = 0.0095, ns) (Figure 2C), suggesting that at 24 hpi, IL27p28 gene expression is mainly dependent on IRF1 activation in CHIKV-infected MDMs. On the other hand, EBI3 mRNA expression had a significant positive correlation with NF-κB1 mRNA (*R* = 0.697, *p* = 00,007), with a high expression at 6 hpi (Figure 2B), suggesting that EBI3 gene expression is dependent on NF-κB activation in CHIKV-infected MDMs.

### 3.4 TLR4 Activation in Human Monocytes-Derived Macrophages Triggers the Activation of NF-κB-Complex Which Drives the Expression of NF-κB-Target Genes

To the best of our knowledge, activation of NF-κB has not been described in MDMs infected with CHIKV. Our RNA-Seq data of CHIKV-infected MDMs suggests activation of NF-κB, that either alone or together with other transcription factors (such as IRF1) could induce the expression of both IL27 subunits. Considering that the NF-kB pathway is commonly activated in response to pathogens (and inflammatory cytokines), and since the initial stimulus to activate NF-kB is the TLR4 ligand LPS (Sen and Baltimore 1986), we reanalyzed publicly available RNA-seq dataset GSE84188 (GEO) (Olejnik et al., 2017), obtained from MDMs stimulated with LPS for 6, 24, or 48 h, to identify possible biomarkers associated with activation NF-kB in macrophages.

**FIGURE 3:**
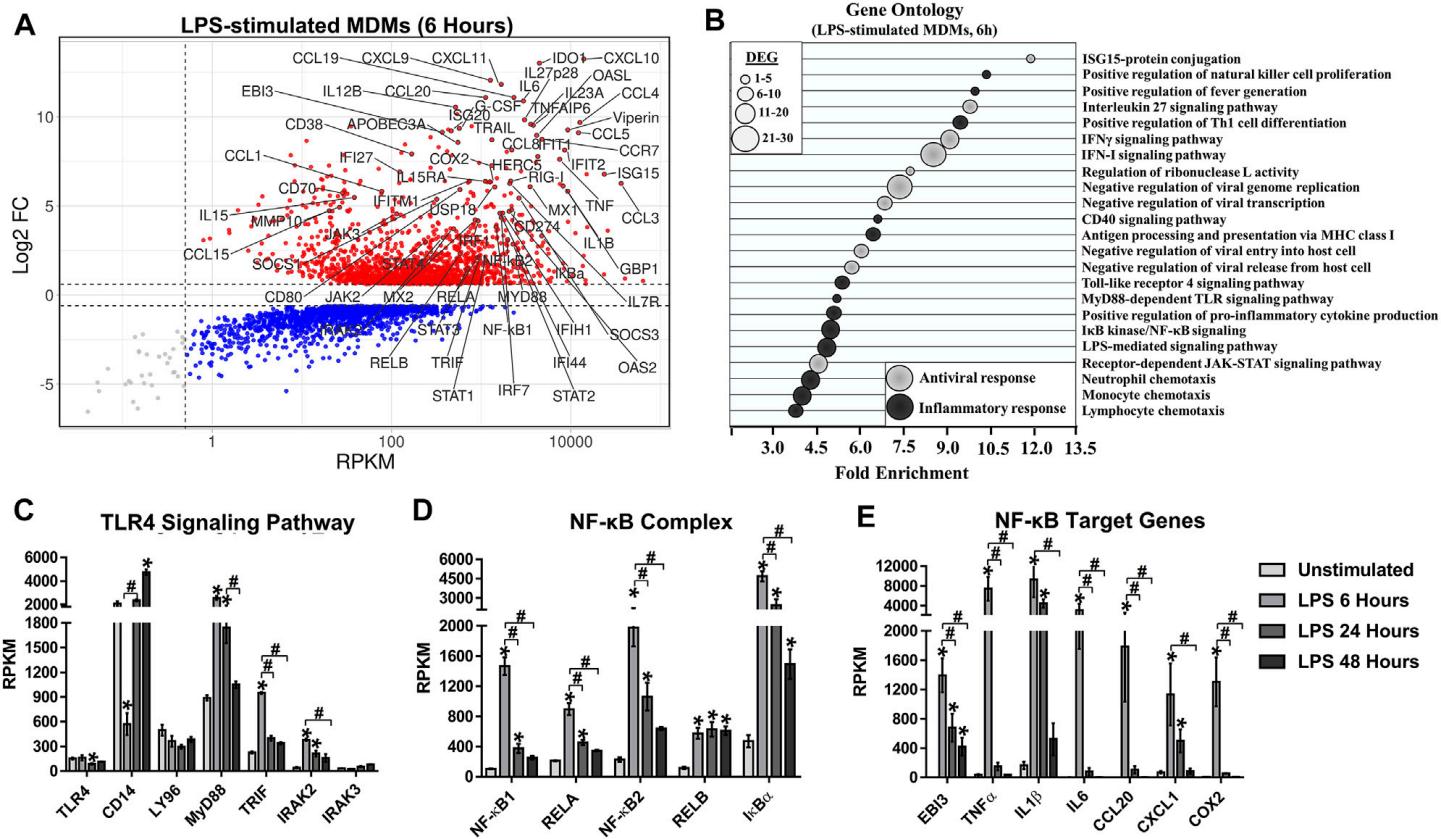
TLR4 activation in human MDMs triggers NF-κB-complex activation which drives the NF-κB-target genes expression. We reanalyzed a publicly available RNA-seq dataset [(GSE84188 (GEO) (Olejnik et al., 2017)], obtained from human MDMs stimulated with 100 ng/ml of LPS, for 6, 24 or 48 h. MA plot of DEG **(A)** and GO enrichment analysis **(B)** from DEG regulated in LPS-stimulated MDMs at 6 h and. DEG in LPS-stimulated MDMs was selected from genes with an FDR< 0.05 and |Log2 Fold Change (LPS-stimulated MDMs/unstimulated MDMs |> 0.6. To quantify mRNAs abundance, we use the Reads per kilobase per million mapped reads (RPKM). *n* = 3. mRNAs abundance (RPKM) of TLR4 signaling pathway components **(C)**, NF-κB-complex components **(D)**, and NF-κB-target genes **(E)** in unstimulated and LPS-stimulated MDMs. Data are presented as the mean ± SEM. Kruskal–Wallis test with Dunn’s post-test was performed. Significant results between unstimulated and LPS-stimulated MDMs are defined as *p* < 0.05 (*), *p* < 0.01 (**) and *p* < 0.002 (***). Significant results between treatment-times comparisons are defined as *p* < 0.05 (#), *p* < 0.01 (##) and *p* < 0.002 (###).

To define top DEGs, we selected genes with an FDR< 0.05 and |Log2 FC (LPS-stimulated MDMs/unstimulated MDMs) |> 0.6. Analysis of the data showed that the peak of the transcriptional response of LPS-stimulated MDMs occurs at 6 h of treatment (1,652 up- and 1,422 down-regulated DEGs) (Figure 3A), and decreases as a function of time (data not shown). As we observed in CHIKV-infected MDMs, the LPS induced genes included pro-inflammatory cytokines, including TNFα, IL1β, IL6, IL12p40, IL15, IL18, TNF-related apoptosis-inducing ligand (TRAIL), and B-cell activating factor (BAFF); both CC-and CXC-chemokines, including CCL1, CCL3, CCL4, CCL5, CCL7, CCL8, CCL15, CCL20, CCL23, CXCL1, CXCL2, CXCL3, CXCL6, CXCL8/IL8, CXCL9, CXCL10, and CXCL11 (Figure 3A). GO functional analysis of DEG was performed to gain an insight into the biological roles of the most significantly up-regulated genes in LPS-stimulated MDMs at 6 h (Figure 3B). As expected and previously reported (Alasoo et al., 2015), DEGs were enriched for biological processes linked with the induction of a robust pro-inflammatory program, including TLR4 signaling pathway, MyD88-dependent TLR signaling pathway, LPS-mediated signaling pathway, IκB kinase/NF-κB signaling, positive regulation of pro-inflammatory cytokine production, positive regulation of fever generation, monocyte, neutrophil and lymphocyte chemotaxis, and others, suggesting that modulation of these genes may be essential for the induction of a robust pro-inflammatory response in LPS-stimulated MDMs.

Transcriptomic analysis of LPS-stimulated MDMs showed that TLR4 activation up-regulated expression of TLR4 signaling components (Figure 3C), including MyD88, TRIF, and IRAK2, with a peak of mRNA expression at 6 h. This peak of mRNA expression overlapped with significant expression of NF-κB components (NF-κB1, NF-κB2, RELA, RELB, and IκBα) (Figure 3D), and NF-κB-target genes (Figure 3E). In addition, NF-κB1 mRNA expression correlated with the expression of NF-κB-target genes including EBI3 (*R* = 0.8312, *p* < 0.0001), TNFα (*R* = 0.8457, *p* < 0.0001), IL1β (*R* = 0.7025, *p* = 0.0007), IL6 (*R* = 0.7558, *p* = 0.0002), CCL20 (*R* = 0.7557, *p* = 0.0002), CXCL1 (*R* = 0.6345, *p* = 0.0019) and COX2 (*R* = 0.8312, *p* < 0.0001) (Supplementary Material S1A). The results suggest that activation of TLR4 by LPS in human MDMs leads to NF-κB activation and drives a robust pro-inflammatory response, as we observed in CHIKV-infected MDMs. Furthermore, those results support our hypothesis that it is possible to predict the activation of NF-κB and expression of NF-κB-target genes through the transcriptional analysis.

### 3.5 Chikungunya Virus Infection in Monocytes-Derived Macrophages Induces the Expression of mRNAs of TLR1/TLR2 Signaling Components, NF-κB-Complex and NF-κB-Target Genes, Including EBI3

**FIGURE 4:**
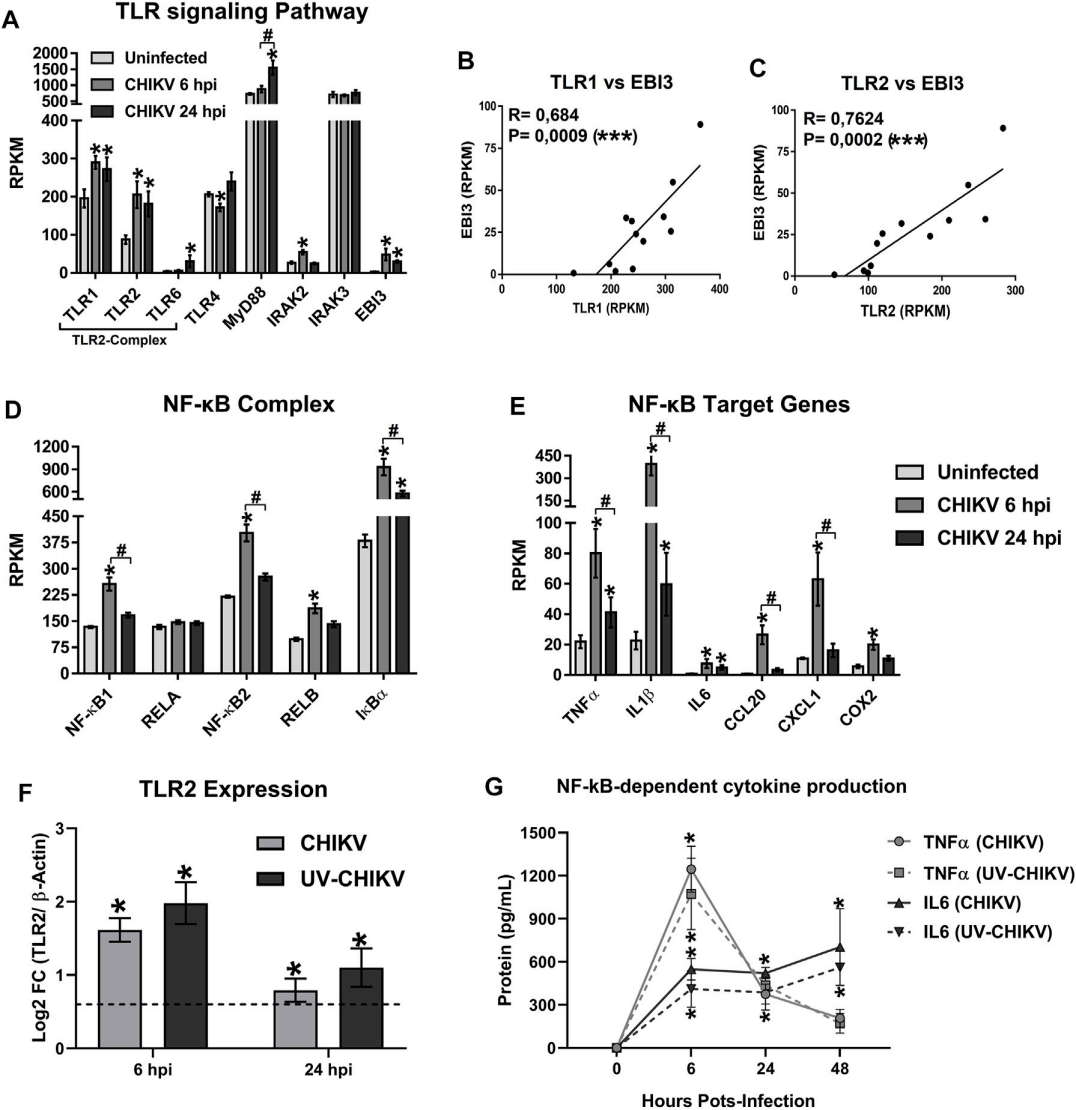
The CHIKV infection of MDMs induces the mRNA expression of TLR1/2 signaling pathway components, NF-κB-complex and EBI3. Human MDMs cultures were left uninfected or infected with CHIKV at MOI 5. Cell lysates were obtained at 6 and 24 hpi, and RNA-seq was performed. mRNAs abundance (RPKM) of TLR signaling pathway components and EBI3 **(A)** in uninfected and CHIKV-infected MDMs. Data are presented as the mean ± SEM. Kruskal–Wallis test with Dunn’s post-test was performed. Significant results between uninfected and CHIKV-infected MDMs are defined as *p* < 0.05 (*), *p* < 0.01 (**) and *p* < 0.002 (***). Significant results between treatment-times comparisons are defined as *p* < 0.05 (#), *p* < 0.01 (##) and *p* < 0.002 (###) *n* = 4. Pearson’s correlation between TLR1 (B) or TLR2 **(C)**, with EBI3 mRNA abundance (RPKM) in uninfected and CHIKV-infected MDMs. Significant results are defined as *p* < 0.05 (*), *p* < 0.01 (**) and *p* < 0.002 (***). mRNA abundance (RPKM) of NF-κB-complex components **(D)**, and NF-κB-target genes **(E)**, in uninfected and CHIKV-infected MDMs. Data are presented as the mean ± SEM. Kruskal–Wallis test with Dunn’s post-test was performed as decrived in **(A)**. Human MDMs cultures were infected at MOI 5 of CHIKV or a non-replicative UV-inactivated CHIKV (UV-CHIKV). TLR2 mRNA expression (Log2 FC) **(F)** and NF-κB-dependent cytokine production (TNFα and IL6) **(G)** were evaluated at 0, 6 and 24 hpi, by RT-qPCR and ELISA, respectively. Data are presented as the mean ± SEM. Kruskal–Wallis test with Dunn’s post-test was performed. Significant results between uninfected and CHIKV- or UV-CHIKV-infected MDMs are defined as *p* < 0.05 (*), *p* < 0.01 (**) and *p* < 0.002 (***). Significant results between CHIKV-infected MDMs and UV-CHIKV-infected MDMs are defined as *p* < 0.05 (#), *p* < 0.01 (##) and *p* < 0.002 (###). *n* = 4.

We reported earlier that CHIKV infection induces the production of pro-inflammatory cytokines in human monocytes and MDMs (Valdés-Lopéz et al., 2020). Here, using the transcriptomic analysis of CHIKV-infected MDMs, we report that CHIKV infection up-regulates the expression of TLR2 signaling pathway genes (Figure 4A), including TLR1, TLR2, and IRAK2, with high mRNA expression at 6 hpi. Furthermore, CHIKV infection also leads to the expression of EBI3 mRNA at 6 hpi (Figure 4A). Interestingly, a significant positive correlation between TLR1 (*R* = 0.684, *p* = 0.0009) (Figure 4B) and TLR2 mRNA (*R* = 0.7624, *p* = 0.0002) (Figure 4C) with EBI3 mRNA in CHIKV-infected MDMs was observed, suggesting that activation of TLR1/2 heterodimer is associated with the expression of EBI3 mRNA. In addition, we noted high levels of mRNA expression of NF-κB components (Figure 4D) and NF-κB-target genes (Figure 4E) in CHIKV-infected MDMs at 6 hpi.

To validate the transcriptomic results relevant to the induction of TLR1/2 signaling and NF-κB-complex activation in CHIKV-infected MDMs, human MDMs cultures were infected with CHIKV wild type or with non-replicative UV-inactivated CHIKV (UV-CHIKV) at MOI 5. and the TLR2 mRNA expression and NF-κB-dependent cytokine production (TNFα and IL6) were evaluated at 6 and 24 hpi, by RT-qPCR and ELISA, respectively. We found that infection of human MDMs with both CHIKV wild type and UV-CHIKV induced a high expression of TLR2 mRNA at 6 hpi, without significant differences between CHIKV- and UV-CHIKV-infected MDMs (Figure 4F). Results were consistent with high and significant production of TNFα and IL6, both induces in response to NF-κB activation, in both CHIKV- and UV-CHIKV-infected MDMs, with peak of proteins production at 6 hpi (Figure 4G). Interestingly, the kinetics of TLR2 mRNA expression and TNFα protein production were consistent with the kinetics of TLR2 and TNFα mRNA expression observed by RNA-seq (Figures 4A,E, respectively), with peak of protein/mRNA expression of TNFα at 6 hpi that decreased as a function of time. In contrast, a sustained high IL6 protein production was observed from 6 to 48 hpi in both CHIKV- and UV-CHIKV-infected MDMs (Figure 4G). Together, our results suggest that the early recognition of CHIKV-PAMPs by TLR1/2 promotes the activation of TLR1/2 signaling pathway leading the activation of NF-κB, and the expression of NF-κB-target genes, including EBI3 mRNA in infected MDMs, in a manner independent of CHIKV replication.

### 3.6 Chikungunya Virus Infection of Monocytes-Derived Macrophages Induces the Expression of mRNAs of TLR3 Signaling Components, IRF1 and IL27p28

**FIGURE 5:**
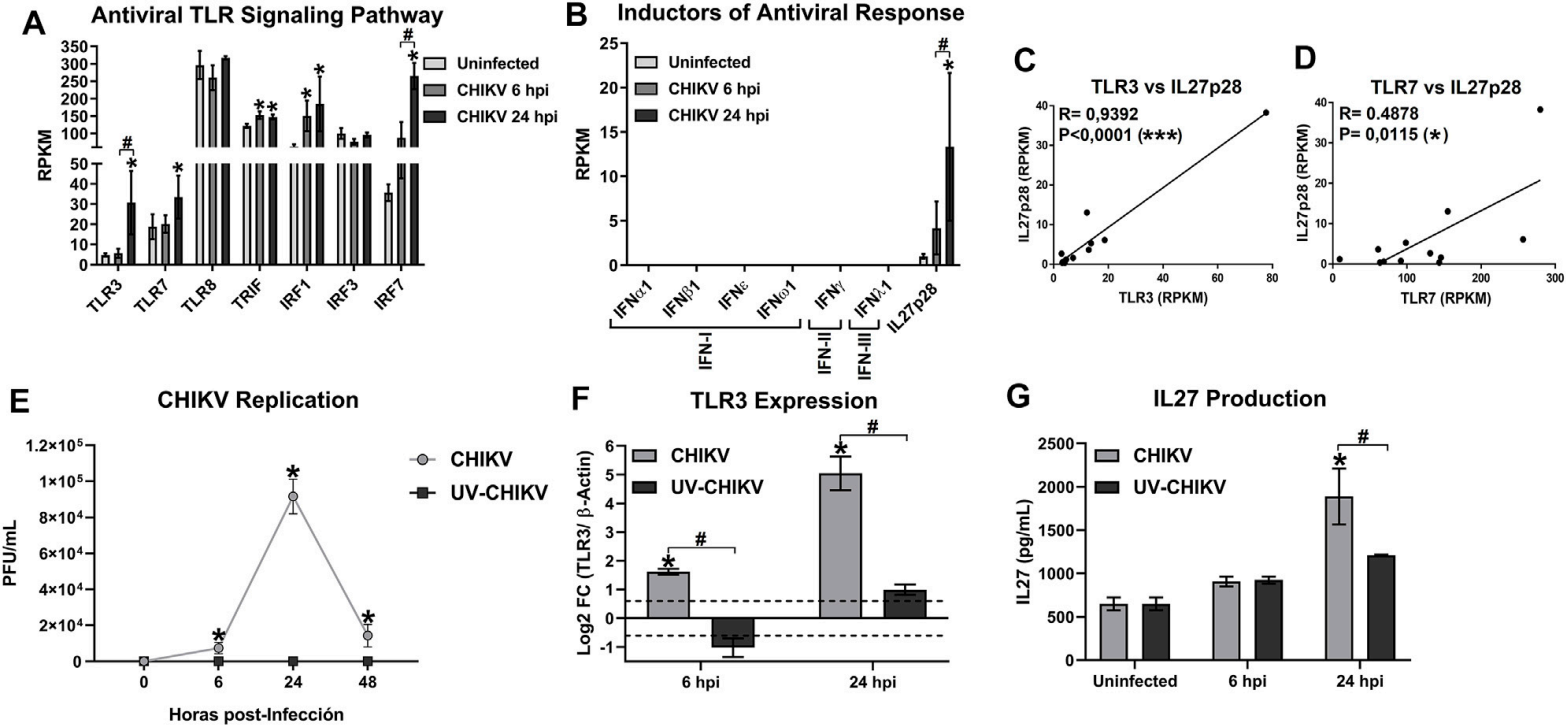
The CHIKV infection of MDMs induces the mRNA expression of TLR3 signaling components, IRF1, and IL27p28. Human MDMs cultures were left uninfected or infected with CHIKV at MOI 5. Cell lysates were obtained at 6 and 24 hpi, and RNA-seq and RT-qPCR were performed. mRNAs abundance (RPKM) of TLR signaling pathway components and IRFs **(A)**, IFNs and IL27p28 **(B)**, in uninfected and CHIKV-infected MDMs. Data are presented as the mean ± SEM. Kruskal–Wallis test with Dunn’s post-test was performed. Significant results between uninfected and CHIKV-infected MDMs are defined as *p* < 0.05 (*), *p* < 0.01 (**) and *p* < 0.002 (***). Significant results between treatment-times comparisons are defined as *p* < 0.05 (#), *p* < 0.01 (##) and *p* < 0.002 (###). *n* = 4. Pearson’s correlation between TLR3 **(C)** or TLR7 **(D)**, with IL27p28 mRNA abundance (RPKM), in uninfected and CHIKV-infected MDMs. Significant results are defined as *p* < 0.05 (*), *p* < 0.01 (**) and *p* < 0.002 (***). Human MDMs cultures were infected at MOI 5 of CHIKV or a non-replicative UV-inactivated CHIKV (UV-CHIKV). CHIKV replication **(E)** was measure by plaque assay on vero cells at 0, 6, 24 and 48 hpi. TLR3 mRNA expression (Log2 FC) **(F)** and IL27 cytokine production **(G)** were evaluated at 0, 6 and 24 hpi, by RT-qPCR and ELISA, respectively. Data are presented as the mean ± SEM. Kruskal–Wallis test with Dunn’s post-test was performed. Significant results between uninfected and CHIKV- or UV-CHIKV-infected MDMs are defined as *p* < 0.05 (*), *p* < 0.01 (**) and *p* < 0.002 (***). Significant results between CHIKV-infected MDMs and UV-CHIKV-infected MDMs are defined as *p* < 0.05 (#), *p* < 0.01 (##) and *p* < 0.002 (###). *n* = 4.

A positive correlation between TLR3 mRNA and AVPs expression was reported in CHIKV-infected MDMs (Valdés-Lopéz et al., 2020), suggesting that TLR3 activation is implicated in the induction of antiviral response. In the current study, using transcriptomic analysis of CHIKV-infected MDMs, we show that CHIKV infection up-regulates the expression of TLRs involved in “sensing” viral RNAs, including TLR3 and TLR7, and components of TLR3 signalings, such as TRIF at 24 hpi (Figure 5A). Furthermore, CHIKV infection-induced IRF1 and IRF7 expression but not IRF3 (Figure 5A). As the expression level of mRNA is consistent with CHIKV replication kinetics (Figure 1A), we suggest that TLR3 and TLR7 expression may be dependent on CHIKV replication (production of viral ssRNA and dsRNA).

As we previously reported (Valdés-López et al., 2021), we observed that CHIKV infection abrogated the expression of all types of IFNs in human MDMs at 6 and 24 hpi (Figure 5B). However, CHIKV-infected MDMs induced IL27p28 mRNA expression, with higher levels at 24 hpi (Figure 5B). Interestingly, IL27p28 mRNA expression has significant positive correlation with TLR3 mRNA (*R* = 0.9392, *p* < 0.0001) (Figure 5C); and a low but significant positive correlation with TLR7 mRNA (*R* = 0.4878, *p* = 0.0115) (Figure 5D).

To validate the transcriptomic results relevant to the activation of TLR3 signaling and induction of IL27 in CHIKV-infected MDMs, human MDMs cultures were infected with CHIKV wild type or UV-CHIKV at MOI 5. TLR3 mRNA expression and IL27 cytokine production were evaluated at 6 and 24 hpi, by RT-qPCR and ELISA, respectively. We quantified the release of infective viral particles in culture supernatants of both CHIKV- and UV-CHIKV-infected MDMs at 6, 24 and 48 hpi, by plaque assay on Vero cells. Unlike to CHIKV wild type, UV-CHIKV was unable to produce an infective viral progeny in human MDMs (Figure 5E). Results were consistent with low induction of TLR3 mRNA expression in UV-CHIKV-infected MDMs, compared with CHIKV-infected MDMs (Figure 5F). Furthermore, UV-CHIKV-infected MDMs do not induce a significant production of IL27 protein in culture supernatants, as compared with uninfected- and CHIKV-infected MDMs at 24 hpi. Our results suggest that induction of TLR3 mRNA expression and IL27 protein production in CHIKV-infected MDMs is dependent on active CHIKV replication, unlike TLR1/2 signaling and NF-kB-complex activation at 6 hpi (Figures 4F,G). Together, these results suggest that recognition of intermediates of CHIKV replication (such as dsRNA), activates the TLR3 signaling pathway to induces IRF1 activation and IL27p28 mRNA expression in infected MDMs, in a IFN-independent manner.

### 3.7 Chikungunya Virus-Infected Monocytes-Derived Macrophages Trigger IL27 Signaling Which Induces Robust STAT1-Dependent Pro-inflammatory and Antiviral Response

**FIGURE 6:**
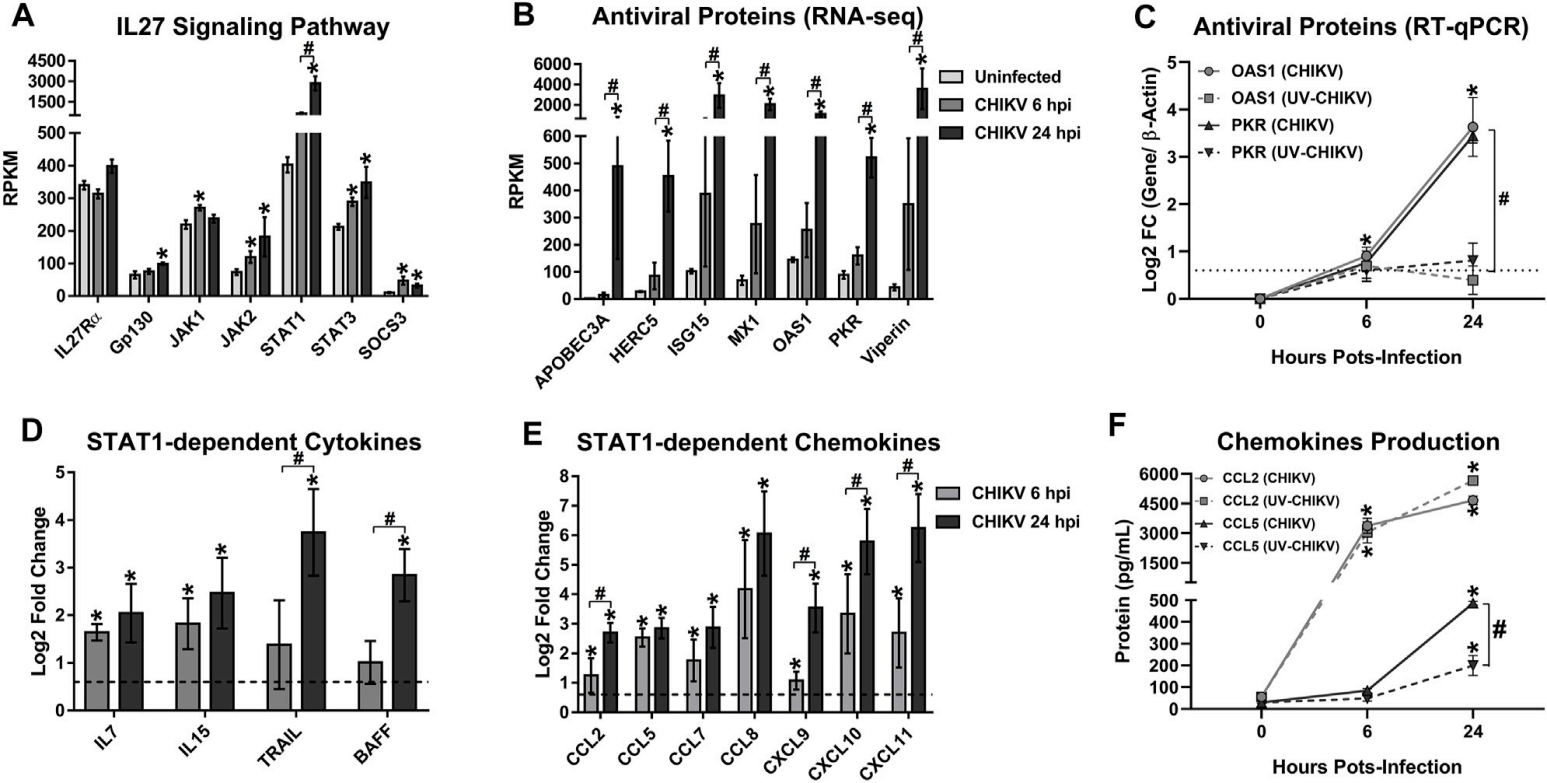
CHIKV-infected MDMs trigger a robust STAT1-dependent pro-inflammatory and antiviral response. Human MDMs cultures were left uninfected or infected with CHIKV at MOI 5. Cell lysates were obtained at 6 and 24 hpi, and RNA-seq was performed. mRNA abundance (RPKM) of IL27 signaling pathway components **(A)** and AVPs **(B)**, in uninfected and CHIKV-infected MDMs. Data are presented as the mean ± SEM. Kruskal–Wallis test with Dunn’s post-test was performed. Significant results between uninfected and CHIKV-infected MDMs are defined as *p* < 0.05 (*), *p* < 0.01 (**) and *p* < 0.002 (***). Significant results between treatment-times comparisons are defined as *p* < 0.05 (#), *p* < 0.01 (##) and *p* < 0.002 (###). Differentially expressed (Log2 FC) STAT1-dependent cytokines **(D)**, CC–and CXC-chemokines **(E)**, in CHIKV-infected MDMs. DEG was defined as genes with an FDR< 0.05 and |Log2 Fold Change (CHIKV-infected MDMs/Uninfected MDMs) |> 0.6. Significant results are defined as *p* < 0.05 (*), *p* < 0.01 (**) and *p* < 0.002 (***). n = 4. Human MDMs cultures were infected at MOI 5 of CHIKV or a non-replicative UV-inactivated CHIKV (UV-CHIKV). AVPs (OAS1 and PKR) mRNA expression (Log2 FC) **(C)**, and STAT1-dependent CC-chemokines production (CCL2 and CCL5) **(F)** were evaluated at 0, 6 and 24 hpi, by RT-qPCR and ELISA, respectively. Data are presented as the mean ± SEM. Kruskal–Wallis test with Dunn’s post-test was performed. Significant results between uninfected and CHIKV- or UV-CHIKV-infected MDMs are defined as *p* < 0.05 (*), *p* < 0.01 (**) and *p* < 0.002 (***). Significant results between CHIKV-infected MDMs and UV-CHIKV-infected MDMs are defined as *p* < 0.05 (#), *p* < 0.01 (##) and *p* < 0.002 (###). *n* = 4.

In our earlier study, the kinetics of IL27p28 mRNA expression and IL27 protein production in CHIKV-infected MDMs was shown to correlate with AVPs expression, in an IFN-independent manner (Valdés-López et al., 2021). Earlier we reported that CHIKV infection-induced IL27 expression; here we now explore if the virus infection also triggers the expression of components of IL27 signaling. As shown in Figure 6A, CHIKV infection up-regulated IL27 signaling components in MDMs. All, except IL27Rα, were up-regulated at 6 and/or 24 hpi, with a high expression of JAK2, STAT1 and STAT3 mRNA at 24 hpi, and that of JAK1 and SOCS3, at 6 hpi. In parallel, we observed high expression of genes at 24 hpi that encode by AVPs (Figure 6B). Further, we observed significant positive correlation of STAT1 mRNA and AVPs mRNA, including APOBEC3A (*R* = 0.6229, *p* = 0.0023), HERC5 (*R* = 0.9491, *p* < 0.0001), ISG15 (*R* = 0.8348, *p* < 0.0001), MX1 (*R* = 0.9528, *p* < 0.0001), OAS1 (*R* = 0.9624, *p* < 0.0001), PKR (*R* = 0.9741, *p* < 0.0001) and Viperin (*R* = 0.9018, *p* < 0.0001) (Supplementary Material S1B). Altogether, these results suggest that the expression of IL27 signaling components, including STAT1, leads to AVPs expression in the absence of IFN types.

Next, we validated transcripts related to AVPs expression by RT-qPCR in CHIKV- and UV-CHIKV-infected MDMs, at 6 and 24 hpi. As shown in Figure 6C, CHIKV-infected MDM expresses significant increases levels of OAS1 and PKR mRNA expression as compared with UV-CHIKV-infected MDMs, and these results linked with our transcriptomic analysis (Figure 5E). Further, unlike to CHIKV wild type, UV-CHIKV infection was unable to induce the expression of AVPs in human MDMs at 24 hpi. These results suggest that induction of AVPs in CHIKV-infected MDMs, like TLR3 mRNA expression (Figure 5F) and IL27 protein production (Figure 5G), is dependent on active viral replication in infected MDMs.We reported earlier that stimulation of THP-1-derived macrophages (with recombinant-human IL27) activated JAK-STAT signaling and induced expression of STAT1-dependent pro-inflammatory factors, including cytokines such as IL7, IL15, TRAIL, and BAFF; CC- and CXC- chemokines, including CCL2, CCL5, CCL7, CCL8, CXCL9, CXCL10, and CXCL11 (Valdés-López et al., 2021). Here, we show that CHIKV infection of MDMs up-regulates STAT1-dependent pro-inflammatory factors, including cytokines (Figure 6D); CC- and CXC-chemokines (Figure 6E), with a high level expression of mRNA at 24 hpi. In addition, we observed that expression of STAT1 mRNA correlated with that of STAT1-dependent cytokines (Supplementary Material S1C), including IL7 (*R* = 0.8893, *p* < 0.0001), IL15 (*R* = 0.7197, *p* = 0.0005), TRAIL (*R* = 0.7345, *p* = 0.0004) and BAFF (*R* = 0.8945, *p* < 0.0001); STAT1-dependent CC-chemokines (Supplementary Material S1C), including CCL2 (*R* = 0.9362, *p* < 0.0001), CCL5 (*R* = 0.6095, *p* = 0.0027), CCL7 (*R* = 0.7019, *p* = 0.0005) and CCL8 (*R* = 0.7019, *p* < 0.0001). STAT1-dependent CXC-chemokines (Supplementary Material S1C), including CXCL9 (*R* = 0.8335, *p* < 0.0001), CXCL10 (*R* = 0.7787, *p* < 0.0001) and CXCL11 (*R* = 0.6726, *p* < 0.0001).

To validate the results, we quantified CCL2 and CCL5 levels in CHIKV- and UV-CHIKV-infected MDMs culture supernatants, by ELISA. We observed the highest levels of both proteins at 24 hpi in both CHIKV- and UV-CHIKV-infected MDMs (Figure 6F), consistent with the kinetics of CCL2 and CCL5 mRNAs expression observed by RNA-seq (Figure 6E). Further, CCL5 levels were higher in CHIKV-infected MDMs than UV-CHIKV-infected MDMs at 24 hpi. Results support the conclusion that activation of IL27 signaling induces STAT1-dependent pro-inflammatory response in CHIKV-infected MDMs. Based on our transcriptomic analysis of CHIKV-infected MDMs, we hypothesize that induction of IL27-dependent pro-inflammatory and antiviral response is dependent on dual signaling pathways: an early signal dependent on recognition of CHIKV-PAMPs by TLR1/2 heterodimer (MyD88-dependent TLRs) to activate NF-κB-complex that induces EBI3 mRNA expression; and a TLR3 (TRIF-dependent TLR) signaling based on the recognition of CHIKV replication intermediates (dsRNA), to activate IRF1 and induce IL27p28 mRNA expression. Thus, synergistic between TLR1/2-MyD88 and TLR3-TRIF signaling triggers the expression of the IL27 subunits in CHIKV-infected MDMs.

### 3.9 TLR1/2-MyD88 Signaling Induces Robust NF-κB-Dependent Pro-Inflammatory Response and Low IL27-Dependent Antiviral Response in Murine Bone Marrow-Derived Macrophages

Our results suggest that CHIKV promotes the expression of IL27 subunits and activates the IL27-dependent signaling following viral infection. Since IL27 induction is dependent on the activation of NF-κB which is under the control of TLRs, it seemed likely that expression of IL27 subunits and induction of pro-inflammatory and antiviral response occurs via synergistic effects of TLR1/2-MyD88 and TLR3-TRIF signaling. To investigate this possibility, we set out to determine whether the molecular network found in CHIKV-infected MDMs is also present in macrophages stimulated with specific TLRs agonists. We reanalyzed publicly available microarray GSE89962 (GEO) (Lin et al., 2017) data on BMDM (from C57BL/6 mice) stimulated with Pam3CSK4 and/or Poly (I:C) ligands, for 8 h.

**FIGURE 7:**
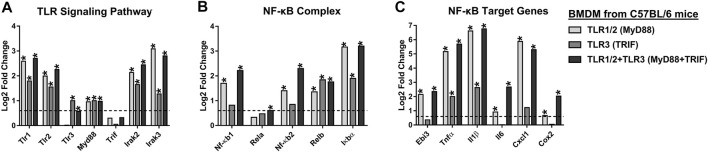
TLR1/2-MyD88, but not TLR3-TRIF signaling, activates NF-κB-complex and induces EBI3 expression in murine BMDM. We reanalyzed the public MicroArray GSE89962 (GEO) (Lin et al., 2017), obtained from BMDM of C57BL/6 mice stimulated with 10 ng/ml Pam3CSK4 and/or 10 μg/ml Poly (I:C), for 8 h. Differentially expressed (Log2 FC) TLR signaling components **(A)**, NF-κB-complex **(B)**, and NF-κB-target genes **(C)**. DEGs were defined as genes with an FDR< 0.05 and |Log2 Fold Change (TLR-treated BMDM/unstimulated BMDM) |> 0.6. Significant results are defined as *p* < 0.05 (*). *n* = 3.

**FIGURE 8:**
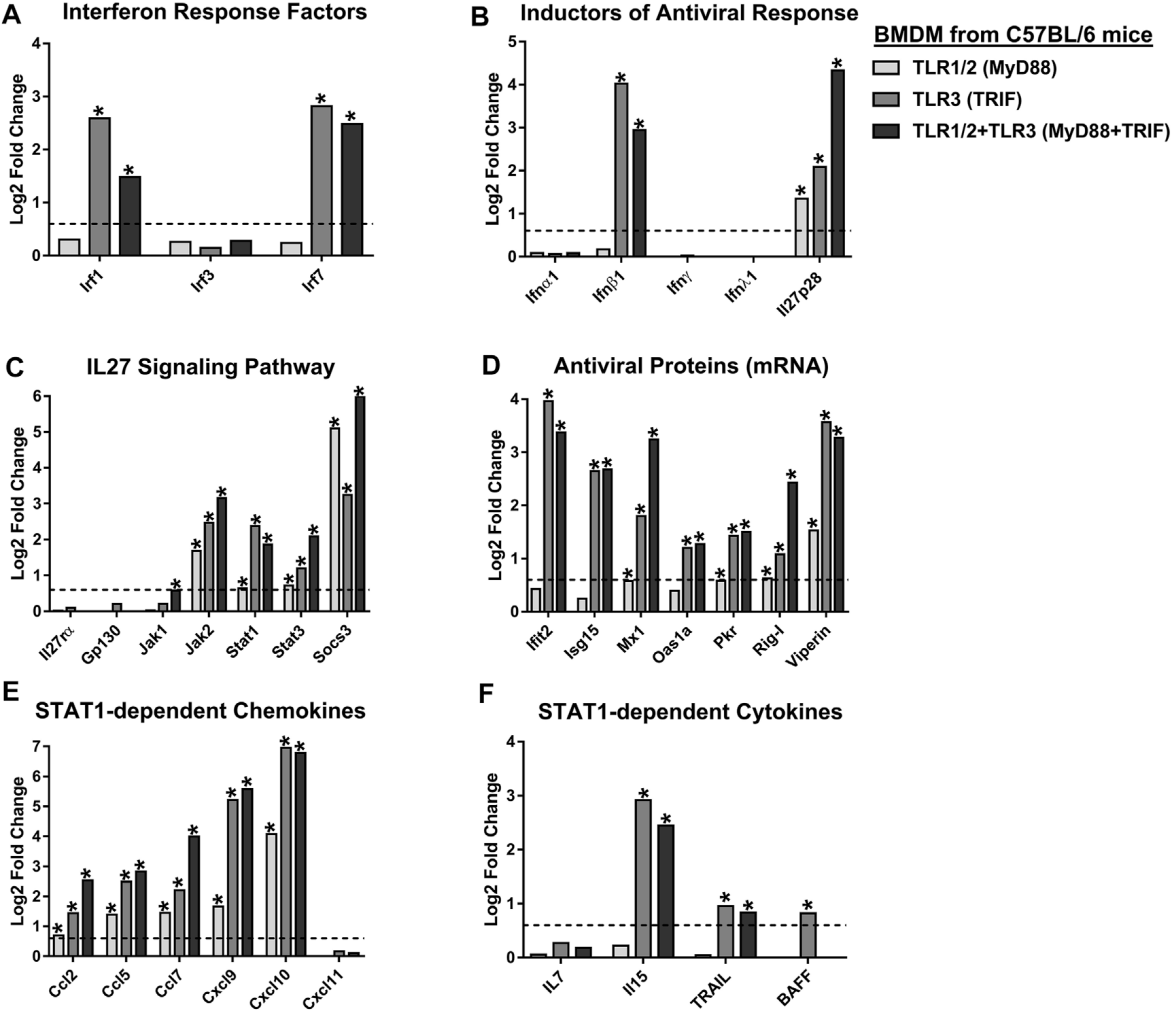
The synergic effect between TLR1/2-MyD88 and TLR3-TRIF triggers the expression of IL27, which induces STAT1-dependent pro-inflammatory and antiviral response in murine BMDM. We reanalyzed the public MicroArray GSE89962 (GEO) (Lin et al., 2017), obtained from BMDM of C57BL/6 mice stimulated with 10 ng/ml Pam3CSK4 and/or 10 μg/ml Poly (I:C), for 8 h. Differentially expressed (Log2 FC) IRFs **(A)**, inductors of antiviral response **(B)**, IL27 signaling components **(C)**, AVPs **(D)**, STAT1-dependent CC- and CXC-chemokines **(E)**, and STAT1-dependent cytokines **(F)**. DEGs were defined as genes with an FDR< 0.05 and |Log2 Fold Change (TLR-treated BMDM/unstimulated BMDM) |> 0.6. Significant results are defined as *p* < 0.05 (*). *n* = 3.

As shown in Figure 7A, transcriptomic analysis shows that Pam3CSK4-treated BMDM up-regulates expression of TLR1/2-MyD88 signaling components, including Tlr1, Tlr2, Myd88, Irak2, and Irak3. These results overlapped with significant expression of NF-κB-complex (Nf-κb1, Nf-κb2, Relb, and IκBα) (Figure 7B), and NF-κB-target genes, including Ebi3, Tnfα, Il1β, Il6, Cxcl1 and Cox2 (Figure 7C). However, activation of TLR1/2-MyD88 does not induce IRFs, including Irf1, Irf3 and Irf7 (Figure 8A), or IFNs (Figure 8B). In addition, activation of TLR1/2-MyD88 in murine BMDM induced low but significant expression of Il27p28 (Figure 8B). Furthermore, the treatment of BMDM with Pam3CSK4 significantly elevated the IL27 signaling components, including Jak2, Stat1, Stat3, and Socs3 (Figure 8C); the ISGs mRNA encoding AVPs (Mx1, Pkr, Rig-I, and Viperin) (Figure 8D), as well as STAT1-dependent CC- and CXC-chemokines (Ccl2, Ccl5, Ccl7, Cxcl9 and Cxcl10) (Figure 8E), but not STAT1-dependent cytokines (Figure 8F). Thus, the activation of TLR1/2-MyD88 in murine BMDM is sufficient to activate NF-κB-complex and induce NF-κB-target genes, including Ebi3. Furthermore, activation of TLR1/2-MyD88 signaling induces a low but significant IL27-dependent pro-inflammatory and antiviral response in murine BMDM.

### 3.10 TLR3-TRIF Signaling Induces Low NF-κB-dependent Pro-inflammatory Response and Robust IFNβ1-dependent Antiviral Response in Murine Bone Marrow-Derived Macrophages

To investigate the impact of TLR3-TRIF signaling, we reanalyzed MicroArray data GSE89962 (Lin et al., 2017), of Poly (I:C) stimulated BMDM, and noted induced expression of both Tlr3 and Myd88 (Figure 7A). Compared to TLR1/2-MyD88 activation, the TLR3-TRIF showed lower but significant up-regulation of Irak2 and Irak3 mRNA (Figure 7A). TLR3-TRIF signaling does not appear to induce NF-κB components (Nf-κb1, Nf-κb2, Rela) (Figure 7B), or NF-κB-target genes, including Ebi3, Il6, Cxcl1 and Cox2 (Figure 7C). These results suggest that TLR3-TRIF activation of NF-κB has an insignificant effect on Ebi3 mRNA expression. However, activation of TLR3-TRIF in murine BMDM induced the expression of IRFs (Irf1 and Irf7, but not Irf3) (Figure 8A); as well, it induced the expression of Il27p28 mRNAs (Figure 8B). Furthermore, the activation of TLR3-TRIF signaling induced significant expression of Ifnβ1 (Figure 8B), JAK-STAT signaling components (Jak2, Stat1, Stat3, and Socs3) (Figure 8C), as well as ISGs mRNA encoding AVPs, such as Ifit2, Isg15, Mx1, Oas1a, Pkr, Rig-I and Viperin (Figure 8D). Similarly, activation of TLR3 leads to an increase in both STAT1-dependent CC- and CXC- chemokines, including Ccl2, Ccl5, Ccl7, Cxcl9 and Cxcl10 (Figure 8E); and STAT1-dependent cytokines (Il15, TRAIL and BAFF) (Figure 8F). Together, results suggest that activation of TLR3-TRIF in murine BMDM induced robust STAT1-dependent pro-inflammatory and antiviral response in an Ifnβ1-dependent manner. However, activation of TLR3-TRIF, by itself, is not sufficient to induce the expression of both IL27 subunits, suggesting that a second signal is required to activate NF-κB-complex and the expression of EBI3. This second signal could be possible by activation of TLR1/2, a MyD88-dependent TLR.

### 3.11 Synergistic Effects of TLR1/2-MyD88 and TLR3-TRIF Trigger IL27 Expression That Induces a Pro-inflammatory and Antiviral Response in Murine Bone Marrow-Derived Macrophages

To better understand the effects of TLR1/2-MyD88 and TLR3-TRIF on the expression of IL27 subunits and the induction of pro-inflammatory and antiviral response, we reanalyzed publicly available microarray data, GSE89962 (Lin et al., 2017), on BMDM treated with Pam3CSK4 and Poly (I:C) ligands (for 8 h). As shown in Figure 7A, co-activation of TLR1/2-MyD88 and TLR3-TRIF up-regulated expression of signaling components of both TLRs, including Tlr1, Tlr2, Tlr3, Myd88, Irak2, and Irak3. This was linked with a high and significant expression of NF-κB-complex components (Figure 7B) and NF-kB-target genes, including Ebi3 (Figure 7C). Co-activation of TLR1/2-MyD88 and TLR3-TRIF also induced the expression of IRFs (Irf1 and Irf7, but not Irf3) (Figure 8A), as well as Il27p28 (Figure 8B). Taken together, results suggest that co-activation of TLR1/2-MyD88 and TLR3-TRIF drive the expression of both IL27 subunits. However, Ifnβ1 expression in TLR1/2 and TLR3 co-stimulated BMDM was lower than TLR3-stimulated BMDM (Figure 8B). We did not observe the expression of Ifnα, Ifnγ and Ifnλ mRNA with either stimulus (Figure 8B).

Further, co-activation of TLR1/2-MyD88 and TLR3-TRIF induced expression of IL27 signaling components (Jak2, Stat1, Stat3, and Socs3) (Figure 8C), as in TLR3-stimulated BMDM. Similarly, co-stimulation of TLR1/2 and TLR3 also induced the expression of genes involved in the antiviral response, including ISGs that encode AVPs (Figure 8D). Thus, co-stimulation leads to expression of STAT1-dependent CC- and CXC- chemokines (Figure 8E), as well as STAT1-dependent cytokines (Il15 and TRAIL) (Figure 8F). Altogether, our results show a synergic effect of TLR1/2-MyD88 and TLR3-TRIF signaling to activate NF-κB-complex and IRF1, that respectively induces the expression of EBI3 and IL27p28 subunits and triggers robust IL27-dependent pro-inflammatory and antiviral response in murine BMDM.

### 3.12 TLR4 Activation in Human and Murine Macrophages Induces IL27 Expression and Strong STAT1-dependent Pro-inflammatory and Antiviral Response

In addition to NF-κB-dependent pro-inflammatory factors, early we observed that activation of TLR4 by LPS in human MDMs up-regulates expression of genes for a high diversity of AVPs (Figure 3A), including APOBEC3-family of proteins, GBP-family of proteins, IFI-family proteins, IFITM-family proteins, OAS-family proteins, ISG15, ISG20, MX1, MX2, PKR, Viperin, and others. Further, GO functional analysis of DEG in LPS-stimulated MDMs at 6 h (Figure 3B) showed enriched for biological processes associated with the induction of a robust antiviral program linked with IL27 signaling pathway, IFNγ, and IFN-I signaling pathway, regulators of ribonuclease L activity, negative regulation of viral genome replication, receptor-dependent JAK-STAT signaling pathway among others (Figure 3B). The results suggest that in addition to a robust MyD88-NF-kB-dependent pro-inflammatory response (Figure 3), TLR4 activation also induces a robust antiviral response in human MDMs.

**FIGURE 9:**
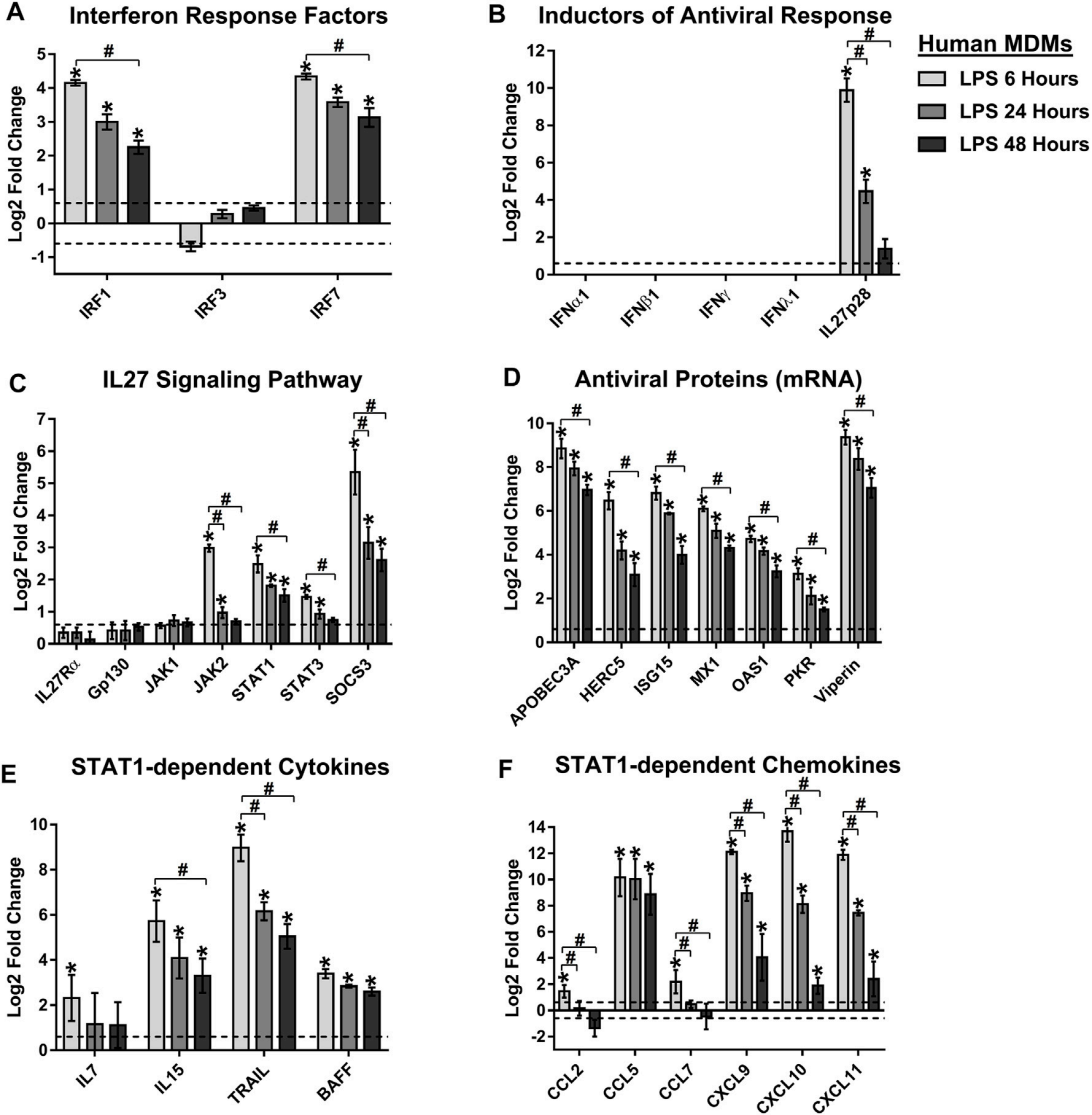
TLR4 activation triggers the expression of IL27, which induces STAT1-dependent pro-inflammatory and antiviral responses in human MDMs. We reanalyzed a publicly available RNA-seq dataset [GSE84188 (GEO) (Olejnik et al., 2017)], obtained from human MDMs stimulated with 100 ng/ml of LPS, for 6, 24 or 48 h. Differentially expressed IRFs **(A)**, inductors of antiviral response **(B)**, IL27 signaling pathway components **(C)**, AVPs **(D)**, STAT1-dependent cytokines **(E)**, and STAT1-dependent CC- and CXC-chemokines **(F)**. Data are presented as the mean ± SEM. Kruskal–Wallis test with Dunn’s post-test was performed. Significant results between unstimulated and LPS-stimulated MDMs are defined as *p* < 0.05 (*), *p* < 0.01 (**) and *p* < 0.002 (***). Significant results between treatment-times comparisons are defined as *p* < 0.05 (#), *p* < 0.01 (##) and *p* < 0.002 (###).

Based on these results, and since TLR4 is the only TLR that signals through both MyD88 and TRIF (Shen et al., 2008; Rajaiah et al., 2015), we hypothesize that TLR4 induces IL27 expression and activates both pro-inflammatory and antiviral response in MDMs (in an IL27-dependent manner). To explore this hypothesis, we reanalyzed publicly available RNA-seq data GSE84188 (GEO) (Olejnik et al., 2017), from human MDMs stimulated with LPS. Transcriptomic analysis shows that TLR4 activation induced high and significant expression of IRF1 and IRF7 (Figure 9A), but not IRF3 and consequently, did not induce the expression of IFNs type in human MDMs (Figure 9B). As expected, we found that LPS-stimulated MDMs trigger strong expression of EBI3 and IL27p28 with a high level of mRNA expression at 6 h of stimulation (Figures 3E, 9B, respectively). Similarly, TLR4 activation up-regulated expression of IL27 signaling components (JAK2, STAT1, STAT3, and SOCS3) (Figure 9C) and ISGs mRNA that encode by AVPs (Figure 9D). Furthermore, LPS-stimulated MDMs induce significant expression of STAT1-dependent cytokines (Figure 8E), and STAT1-dependent CC- and CXC- chemokines (Figure 9F), with kinetics for EBI3 and IL27p28 mRNA (Figures 3D, 9B). Similar results were obtained when we reanalyzed publicly available microarray GSE89962 (GEO) (Lin et al., 2017) performed in murine BMDM stimulated with LPS (Supplementary Material S2). Since no IFN types but high levels of ISGs encoding AVPs were observed, we suggested that this activity is triggered in an IL27-dependent manner.

### 3.13 Validation of MicroArray and RNA-Sequence Results by Real-Time Quantitative PCR and ELISA

**FIGURE 10:**
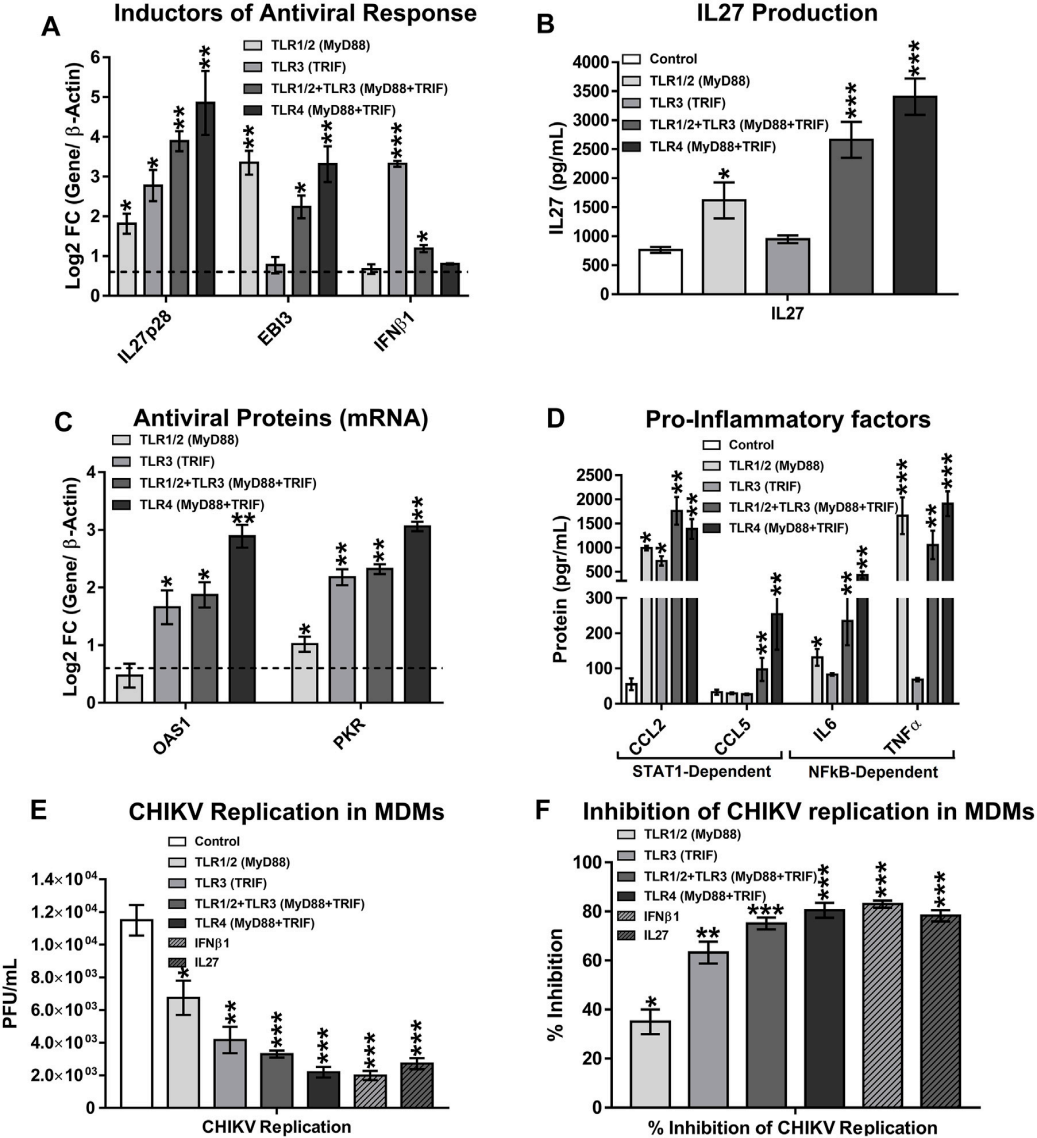
Co-activation of TLR1/2-MyD88 and TLR3-TRIF, or activation of TLR4 triggers expression of IL27 which induces STAT1-dependent pro-inflammatory and antiviral response to control CHIKV replication in human MDMs. Human MDMs cultures were left unstimulated or stimulated with 50 ng/ml Pam3CSK4 and/or 20 μg/ml Poly (I:C), or 50 ng/ml LPS. Cell lysates and culture supernatants were obtained at 8 h, and RT-qPCR and ELISA were performed. mRNA expression of inductors of antiviral response (IL27 and IFNβ1) **(A)**. IL27 accumulation in culture supernatants of MDMs **(B)**. mRNA expression of AVPs (OAS1 and PKR) **(C)**. STAT1-dependent chemokines (CCL2 and CCL5) and NF-κB-dependent cytokines (TNFα and IL6) accumulation in culture supernatants of MDMs **(D)**. Data are presented as the mean ± SEM. Kruskal–Wallis test with Dunn’s post-test was performed. Significant results are defined as *p* < 0.05 (*), *p* < 0.01 (**) and *p* < 0.002 (***). *n* = 4. Human MDMs were pre-treated for 8 h with 50 ng/ml Pam3CSK4 and/or 20 μg/ml Poly (I:C), or 50 ng/ml LPS, or 25 ng/ml of recombinant-human IFNβ1 or IL27. Next, MDMs cultures were infected with CHIKV at MOI 5. Culture supernatants were obtained at 24 hpi and viral titration was performed by plaque assay on Vero cells. CHIKV replication in MDMs **(E)**. Percentage of viral inhibition in MDMs **(F)**. Kruskal–Wallis test with Dunn’s post-test was performed. Significant results between unstimulated and pre-treated MDMs are defined as *p* < 0.05 (*), *p* < 0.01 (**), and *p* < 0.002 (***). *n* = 4.

Next, we validated the expression levels of mRNAs related to IL27 signaling, observed in MicroArray and RNA-Seq results, by RT-qPCR (based on Log2 FC and *p* values) and ELISA. Human MDMs were stimulated with 50 ng/ml Pam3CSK4 and/or 20 μg/ml Poly (I:C) or with 50 ng/ml LPS, for 8 h. As with murine BMDM, we noted that TLR1/2-MyD88 activation induces expression of EBI3 mRNA (Figure 10A), and production of TNFα and IL6 (both NF-κB-dependent cytokines) (Figure 10D). As observed by transcriptomic analysis, activation of TLR1/2-MyD88 induced a low expression of IL27p28 mRNA (Figure 10A) and consequently, low but significant production of IL27 protein in the MDMs stimulated with Pam3CSK4 (Figure 10B). These results are related to low expression of AVPs mRNA (OAS1 and PKR) (Figure 10C) and high production of CCL2, but not CCL5 in MDMs (Figure 10D), in response to TLR1/2-MyD88 activation. Together, results confirm that activation of TLR1/2-MyD88 activated NF-κB-complex and induces the expression of NF-κB-target genes, including EBI3. Furthermore, TLR1/2-MyD88 signaling induced low but significant IL27-dependent pro-inflammatory and antiviral responses in human MDMs.

As observed by transcriptomic data of murine BMDM, activation of TLR3-TRIF signaling in MDMs induced high and significant expression of IL27p28 but low expression of EBI3 mRNA (Figure 10A), suggesting that functional IL27 was insufficient in these cells. The results are consistent with the low production of IL27 protein in culture supernatants of MDMs treated with a TLR3 agonist (Figure 10B). Like murine BMDM, activation of TLR3-TRIF signaling in human MDMs induced significant expression of IFNβ1 (Figure 10A), and ISGs mRNA encoding AVPs (OAS1 and PKR) (Figure 10C). In addition, the production of STAT1-dependent chemokines, such as CCL2, but not CCL5 was found in culture supernatants of MDMs stimulated with Poly (I:C) (Figure 10D). However, like to murine BMDM (Figure 7), low production of NF-κB-dependent cytokines, such as TNFα and IL6 was found in culture supernatants of Poly (I:C)-stimulated MDMs (Figure 10D), suggesting that activation of TLR3 in human and murine macrophages induces low NF-κB activation.

As in murine BMDM, we observed that co-stimulation of TLR1/2-MyD88 and TLR3-TRIF in human MDMs triggers transcription of both IL27 subunits, EBI3 and IL27p28 (Figure 10A), and high production of NF-κB-dependent cytokines (TNFα and IL6) (Figure 10D). These results were consistent with the higher production of IL27 protein in culture supernatants (Figure 10B). However, IFNβ1 expression in TLR1/2 and TLR3-co-stimulated-MDMs was lower than TLR3-stimulated MDMs (Figure 10A), suggesting that co-activation of TLR1/2-MyD88 and TLR3-TRIF signaling in human MDMs activates STAT1-dependent pro-inflammatory and antiviral response through the production of IL27, as was observed in the MicroArray of murine BMDM (Figure 8). Further, we reported a high level of AVPs mRNAs (OAS1 and PKR) (Figure 10C), as well as enhanced production of STAT1-dependent CC- chemokines (CCL2 and CCL5) (Figure 10D) in response to co-activation of TLR1/2-MyD88 and TLR3-TRIF. Similar results were observed in response to TLR4 activation in human MDMs (Figures 10A–F). Altogether, our results confirm that co-activation of TLR1/2 (MyD88 dependent) and TLR3 (TRIF dependent), or the stimulation of TLR4 (MyD88 and TRIF dependent), activated both NF-κB-complex and IRF1 which induces the expression of both subunits of IL27 (EBI3 and IL27p28, respectively), and triggers STAT1-dependent pro-inflammatory and antiviral response (as corroborated in the transcriptomic analysis of human CHIKV-infected MDMs, LPS-stimulated MDMs, and murine TLR-stimulated BMDM).

### 3.14 Toll-Like Receptors Activation Triggers an Antiviral Response and Inhibits Chikungunya Virus Replication in Human Monocytes-Derived Macrophages

To confirm the contribution of TLR1/2, TLR3, and TLR4 activation in inhibiting CHIKV replication, human MDMs were pre-treated for 8 h with 50 ng/ml Pam3CSK4 and/or 20 μg/ml Poly (I:C), or with 50 ng/ml LPS. Recombinant-human IFNβ1 or IL27 were used as positive controls. Next, MDMs were infected with CHIKV (MOI 5), and viral replication was evaluated at 24 hpi. We showed that TLR1/2-MyD88 activation resulted in a significant decrease of CHIKV replication (Figure 10E), with an inhibition of viral replication of 35% ± 5.1 (Figure 10F). The results are consistent with lower production of IL27 protein (Figure 10B), and lower expression of AVPs mRNA compared with other treatments (Figure 10C). On the other hand, TLR3-TRIF activation influenced CHIKV replication (Figure 10E), with inhibition of viral replication of 63.2% ± 4.4 (Figure 10F). Results are consistent with high expression of both IFNβ1 (Figure 10A) and AVPs mRNA (Figure 10C). Co-activation of TLR1/2-MyD88 and TLR3-TRIF, or the activation of TLR4 (MyD88 and TRIF dependent) alone, result in significant attenuation of CHIKV replication (Figure 10E), (inhibition of viral replication of 75.13% ± 2.4 and 80.5% ± 3.1, respectively) (Figure 10F). These results are consistent with the high production of IL27 (Figure 10B) and the expression of AVPs mRNA (Figure 10C), in response to co-activation of TLR1/2 and TLR3, or TLR4 activation. Further, pre-treatment of human MDMs with recombinant-human IFNβ1 or IL27 induces a strong antiviral response that abrogated CHIKV replication in human MDMs (Figure 10E), (inhibition of viral replication of 83% ± 1.5 and 78.2% ± 2.3, respectively) (Figure 10F), confirming that both IFNβ1 and IL27 have comparable effects on inhibition of CHIKV replication in human MDMs. Of note, TLR4 activation triggers a robust antiviral response, similar to IFNβ1 and IL27, that is stronger than TLR3. To the best of our knowledge, this is the first study showing such an antiviral response.

## 4 Discussion

### 4.1 The Interferon-like Antiviral Properties of Interleukin 27

IL27 is a heterodimeric cytokine expressed by antigen-presenting cells (APCs), including activated monocytes, macrophages, and dendritic cells, and has been associated with pro- and anti-inflammatory properties (Wirtz et al., 2005; Hunter and Kastelein 2012; Valdés-López et al., 2021). IL27 enhances IFNγ production by naive CD4^+^ T cells and NK cells (Lucas et al., 2003; Kumar et al., 2019). Further, IL27 induces Th1 and down-regulates Th17 response in CD4^+^ T cells (Cao et al., 2008; Murugaiyan et al., 2009). Additionally, IL27 acts on naïve CD8^+^ T-cells to enhance the generation of cytotoxic T lymphocytes (Morishima et al., 2005), maintain plasmacytoid dendritic cells (pDCs) (Harker et al., 2018), and support T follicular helper cell function (Batten et al., 2010). Furthermore, IL27 induces a strong antiviral response against HIV-1, HBV, HCV, IAV, ZIKV, and CHIKV, in the respective host cell types (Fakruddin et al., 2007; Frank et al., 2010; Liu et al., 2012; Cao et al., 2014; Kwock et al., 2020; Valdés-López et al., 2021). However, molecular bases of IL27 mediated antiviral response is only beginning to be understood. Recombinant-human IL27 activated JAK-STAT signaling pathway in human normal epidermal keratinocytes (HNEKs) and induced expression of AVPs, including OAS1, OAS2, OASL, and MX1, in an IL27Rα- and STAT1-dependent manner, but independent of IFNAR1 and STAT2 (Kwock et al., 2020). Additionally, the authors reported that IL27-treated HNEKs inhibited replication of ZIKV and Sendai virus *in vitro*. Furthermore, using Ifnar1−/−, Il27ra−/−, and Ifnar1−/−/Il27ra−/− mice infected with ZIKV, Kwock et al. (2020) showed that subcutaneous administration of recombinant IL27 reduced mortality and onset of neurological symptoms of ZIKV infection in an IFN-independent manner, confirming the ability of IL27 to induce a protective antiviral response against ZIKV, both *in vitro* and *in vivo*. In agreement with these results, we recently reported that the IL27 pathway is activated in CHIKV-infected MDMs (Valdés-López et al., 2021). We found that kinetics of IL27p28/EBI3 mRNA expression and IL27 protein production correlated with the expression of AVPs. Furthermore, we observed that THP-1-derived macrophages treated with recombinant-human IL27 activated JAK-STAT signaling pathway and induced robust STAT1-dependent pro-inflammatory and antiviral response, comparable to CHIKV-infected MDMs (Valdés-López et al., 2021). Together, these reports demonstrate the Interferon-like antiviral properties of IL27, both *in vitro* and *in vivo*, and suggest that activation of the IL27 signaling pathway by APCs constitutes a recurrent mechanism to induce antiviral state and control viral replication, in an IFN-independent manner.

### 4.2 Regulation of IL27 Expression in Macrophages and Dendritic Cells Is Dependent of Toll-Like Receptors Pathway, NF-κB and IRF1

**FIGURE 11:**
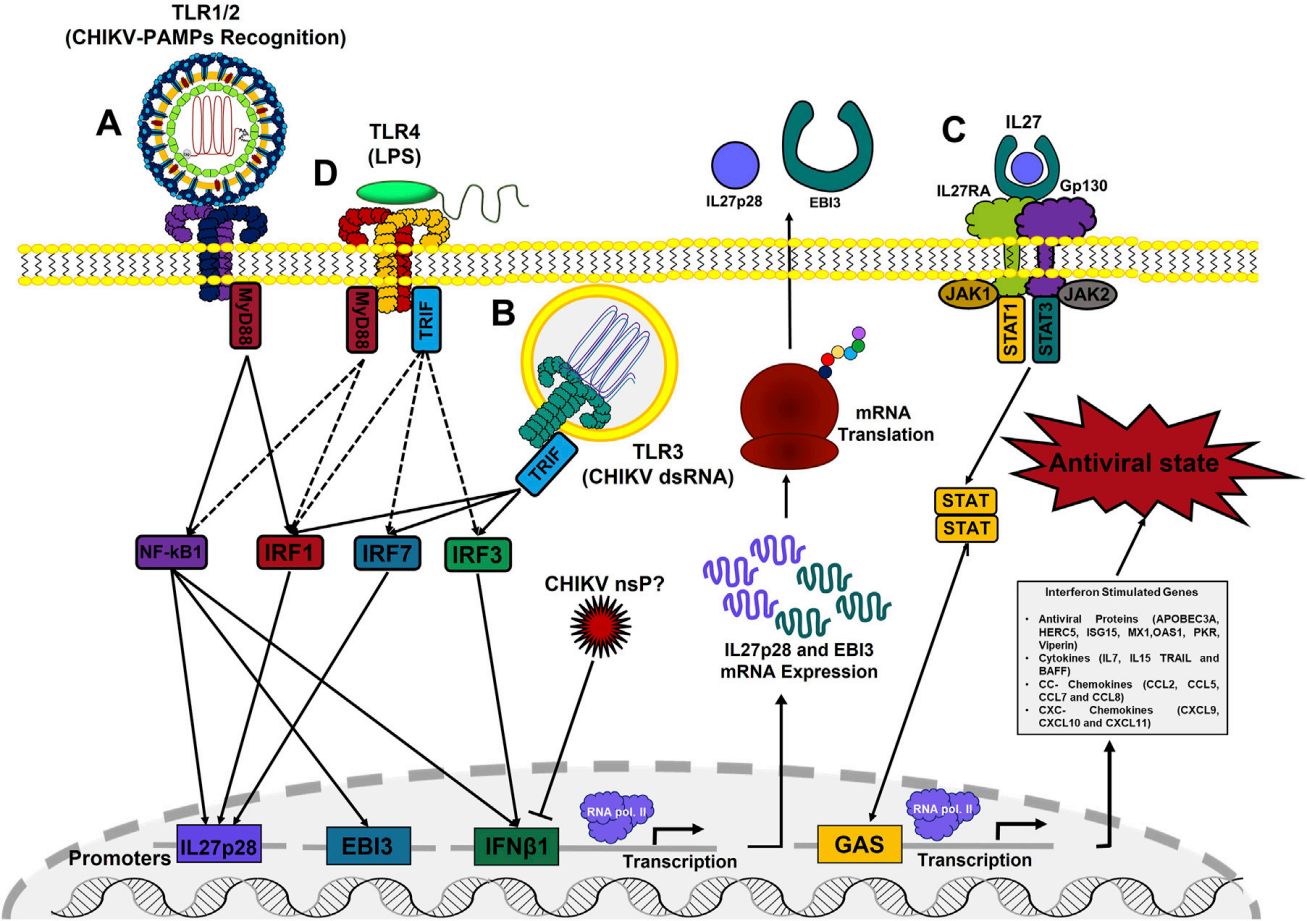
Schematic model of induction of IL27-dependent pro-inflammatory and antiviral response in CHIKV-infected MDMs. IL27 production in CHIKV-infected MDMs is dependent on two signaling pathways depending on the TLRs activation. An early signal dependent on recognition of CHIKV-PAMPs by TLR1/2-MyD88 to activate NF-κB-complex and induce EBI3 mRNA expression **(A)**; and a late signal dependent on recognition of intermediates of CHIKV replication by TLR3-TRIF, to activate IRF1 and induces IL27p28 mRNA expression **(B)**. Both signals are required to produce a functional IL27 protein and activated IL27 signaling pathway involved in the induction of ISGs expression, including AVPs, cytokines, CC- and CXC- chemokines; to control CHIKV replication in human MDMs, in an IFN-independent manner **(C)**. Activation of TLR4, by LPS, induces a high expression of both IL27 subunits in human MDMs and murine BMDM and activated robust STAT1-dependent pro-inflammatory and antiviral response in an IL27-dependent manner **(D)**.

Innate immune cells including macrophages and dendritic cells (DCs), use a variety of TLRs to recognize invading pathogens and elicit an appropriate innate immune response. In this study, we demonstrated that following stimulation of TLR4 (with LPS), or co-stimulation of TLR1/2 and TLR3 (with Pam3CSK4 and Poly I:C, respectively), human MDMs and murine BMDM induce EBI3 and IL27p28 mRNA expression, suggesting that TLR4 and TLR1/2-TLR3 crosstalk dictates IL27 production in macrophages to orchestrate both pro-inflammatory and antiviral response (Figure 11).

In agreement with our observations, Wirtz et al. (2005) reported that stimulation of murine DCs via TLR2, TLR4, and TLR9 (MyD88-dependent TLRs) transactivated the EBI3 promoter via MyD88 and NF-κB. Further, they reported that EBI3 mRNA expression was reduced or abrogated in TLR2, TLR4, TLR9, and MyD88 knockout mice, whereas both basal and inducible EBI3 mRNA levels were strongly suppressed in NF-κB1-deficient mice, suggesting that EBI3 expression in DCs is transcriptionally regulated by TLRs activation via MyD88 and NF-κB signaling pathway. On the other hand, it has been reported that IL27p28 is highly expressed in murine DCs and peritoneal macrophages in response to TLR3 and TLR4 activation (Molle et al., 2007; Bosmann et al., 2012). Furthermore, Zhang et al., 2010 reported that IRF1 and IRF8 regulate IL27p28 gene transcription in murine macrophages in response to TLR4 activation, by specifically binding to the IRF1 response element in the IL27p28 promoter. Altogether, these reports support our hypothesis and confirm that transcriptional expression of IL27 in macrophages and DCs is dependent on two signaling pathways and it depends on the TLRs involved; thus, the activation of NF-κB-complex by a MyD88-dependent TLR to induces the EBI3 mRNA expression, while the activation of IRF1 by a TRIF-dependent TLR to induces the expression of IL27p28 mRNA (Figures 11A–D). Furthermore, based on our analysis of three macrophage transcriptomes, we suggest that regulation of IL27 expression by TLRs and pro-inflammatory and antiviral properties of IL27 signaling are conserved events in the evolution of rodents and primates.

### 4.3 TLR4 as an Inducer of Antiviral Response

TLR4 is the only one among 10 TLRs described in humans that signals through both MyD88 and TRIF (Shen et al., 2008; Rajaiah et al., 2015). Although the activation of TLR4 by LPS has been associated with robust pro-inflammatory response, dependent on MyD88 and NF-κB-complex activation (Küper et al., 2012; Sakai et al., 2017), a possible role of TLR4 signaling in the induction of antiviral response has also been suggested. In the study by Kurt-jones et al. (2000) reported that recognition of respiratory syncytial virus (RSV)-fusion protein by TLR4 and CD14, induced antiviral response and reduced viral persistence in lungs of RSV-infected mice, suggesting a potential role of TLR4 signaling in induction of antiviral response to bacteria and viruses. Toshchakov et al. (2002) reported that murine macrophages (from CH3/Ouj mice) stimulated with the TLR4 agonist *E. coli-*LPS, but not with TLR2 agonists, induced early and transient expression of IFNβ1 mRNA and STAT1 phosphorylation at 2 h of stimulation. However, Das et al. (2018) reported that the transcriptional profile of IFN-γ-primed and unprimed BMDM (from C57BL/6 mice) induced pro-inflammatory and antiviral response to LPS, with high expression of ISGs, including AVPs (Ifit1, Gbp2, Isg15, Mx1, Oas2, Rig-I, and Viperin), and STAT1-dependent pro-inflammatory factors (Il7, Il15, TRAIL, Ccl2, Ccl5, Ccl7, Cxcl9, and Cxcl10), but not IFNs. Furthermore, the authors reported that LPS induced the mRNA expression of transcription factors, such as NF-κB1, IκBα, STAT1, IRF1, IRF7, in addition to IL27p28 mRNA. Additionally, Mlcochova et al.,. 2020 proposed that TLR4 activation in human MDMs can potently induce a SAMHD1-dependent antiretroviral response by an IFN-independent pathway. In the study of Song et al. (2021) reported that TLR4 signaling in human MDMs, but not monocytes, engages transcription factor IRF1, which facilitates the opening of ISGs loci for transcription. In line with the literature, we reported that activation of TLR4 by LPS in both human MDMs and murine BMDM (from C57BL/6 mice), activated both NF-κB-complex and IRF1 to induce the expression of both IL27 subunits (EBI3 and IL27p28, respectively), that induces a robust STAT1-dependent pro-inflammatory and antiviral response (Figure 11D), in an IFN-independent manner.

Altogether, these reports suggest that TLR4 signaling induced a robust MyD88-NF-κB-dependent pro-inflammatory response, and a robust STAT1-dependent pro-inflammatory and antiviral response-dependent on IL27 production. To the best of our knowledge, this is the first reported linking activation of TLR4 signaling by LPS, with the induction of a robust STAT1-dependent pro-inflammatory and antiviral response in macrophages, in an IL27-dependent manner, against CHIKV infection in human MDMs.

### 4.4 Interleukin 27, a “Double-Edged Sword” in Control and Pathogenesis of Chikungunya Virus Infection

Although antiviral properties of IL27 contribute to the control of CHIKV replication, its persistent or unregulated production could have important implications in the immunopathogenesis of CHIKF. Acute and chronic phases of CHIKF have an important immunopathological compound (Revised in (Valdés-López et al., 2019)). Here, we showed that IL27 promotes a protective antiviral state in MDMs to control CHIKV replication. However, IL27 also mediates a high expression of STAT1-dependent pro-inflammatory factors, including cytokines (IL7, IL15, BAFF and TRAIL), CC- and CXC-chemokines (CCL2, CCL5, CCL7, CCL8, CXCL9, CXCL10, and CXCL11). All these cytokines/chemokines, like IL27, are considered arthritogenic factors and are up-regulated in Rheumatoid arthritis patients as well as CHIKV-infected patients, and had been associated with the development of inflammation and joint pain (González-álvaro et al., 2006; Wong et al., 2010; Adamopoulos et al., 2013; Burska et al., 2014; Yap et al., 2018; Kim et al., 2019). Furthermore, Cavalcanti et al., 2019 reported that in patients with chronic symptoms of CHIKF, a significant and positive correlation between IL27 serum levels and tender joints was observed, suggesting a pathogenic role of IL27 in chronic CHIKV-infected patients and a possible role of IL27 in the pathogenesis of CHIKV-dependent arthralgia and arthritis. Altogether, these reports suggest that, despite the antiviral properties of IL27 and its ability to control CHIKV replication, persistent production of IL27 could be associated with induction of persistent STAT1-dependent pro-inflammatory response, leads to joint pain and arthralgias in CHIKV-infected patients, suggesting that IL27 signaling pathway could be a good therapeutic target to control the chronic symptoms of CHIKF in CHIKV-infected patients, and the inflammation and joint pain in Rheumatoid arthritis patients.

### 4.5 Conclusion

The key conclusion from this study is that induction of IL27-dependent pro-inflammatory and antiviral response in CHIKV-infected MDMs is dependent on two signaling pathways: an early signal dependent on recognition of CHIKV-PAMPs by TLR1/2-MyD88 to activate NF-κB-complex and induces EBI3 mRNA expression (Figure 11A); and second signaling dependent on recognition of intermediates of CHIKV replication (such as dsRNA) by TLR3-TRIF, to activate IRF1 and induces IL27p28 mRNA expression (Figure 11B). Both signaling pathways are required to produce a functional IL27 protein and activated IL27 signaling pathway involved in the induction of ISGs expression, including AVPs, cytokines, CC- and CXC- chemokines to control CHIKV replication in human MDMs, in an IFN-independent manner (Figure 11C). Furthermore, we reported that activation of TLR4 by LPS, both human MDMs and murine BMDM, induces a high expression of both subunits of IL27 and triggers a strong IL27-dependent pro-inflammatory and antiviral response, instead IFNs (Figure 11D). Our data also provide important insight into innate immune and antiviral responses and may influence strategies for modulating host immune responses to control CHIKV and other viral infections.

## Data Availability

The datasets presented in this study can be found in online repositories. The names of the repository/repositories and accession number(s) can be found below: NCBI GEO, accession no: GSE193977.
