# Supplementary figures and images for: Synergistic Effects of Toll-Like Receptor 1/2 and Toll-Like Receptor 3 Signaling Triggering Interleukin 27 Gene Expression in Chikungunya Virus-Infected Macrophages
Source: Front Cell Dev Biol. 2022 Feb 9;10:812110. doi: 10.3389/fcell.2022.812110 (PMC8863767; doi:10.3389/fcell.2022.812110)

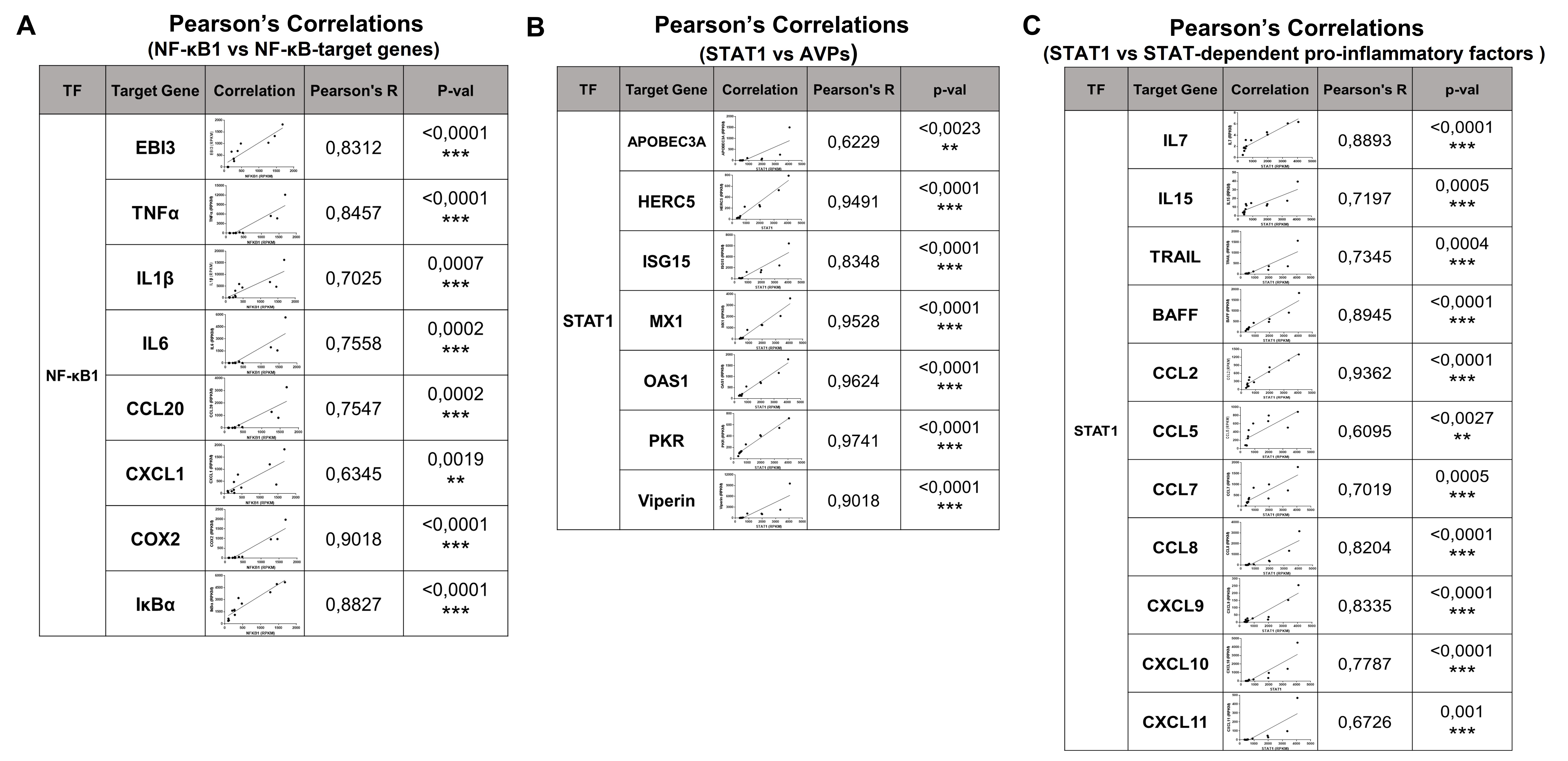

Supplement: Supplementary file 1 [file Image1.JPEG]

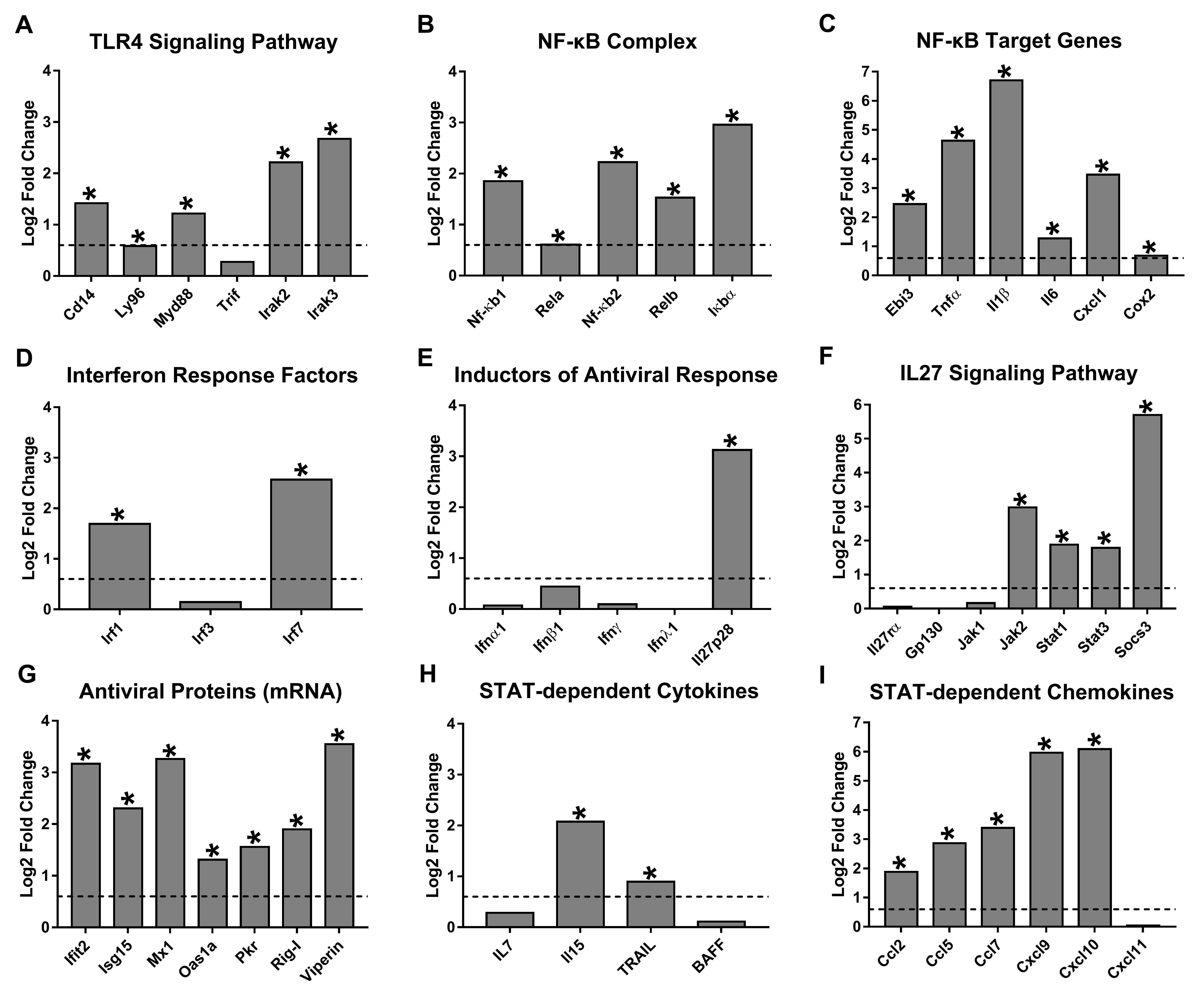

Supplement: Supplementary file 2 [file Image2.JPEG]
